# Advanced Polymeric Nanocomposite Membranes for Water and Wastewater Treatment: A Comprehensive Review

**DOI:** 10.3390/polym15030540

**Published:** 2023-01-20

**Authors:** Abhispa Sahu, Raghav Dosi, Carly Kwiatkowski, Stephen Schmal, Jordan C. Poler

**Affiliations:** 1 American Nano, LLC, 2011 Muddy Creek Road, Clemmons, NC 27012, USA; 2 Department of Chemistry, University of North Carolina at Charlotte, 9201 University City Blvd, Charlotte, NC 28223, USA; 3 Goulston Technologies, 700 N Johnston St, Monroe, NC 28110, USA

**Keywords:** polymer nanocomposites, water treatment, inorganic nanoparticles, desalination, computational studies, biopolymers

## Abstract

Nanomaterials have been extensively used in polymer nanocomposite membranes due to the inclusion of unique features that enhance water and wastewater treatment performance. Compared to the pristine membranes, the incorporation of nanomodifiers not only improves membrane performance (water permeability, salt rejection, contaminant removal, selectivity), but also the intrinsic properties (hydrophilicity, porosity, antifouling properties, antimicrobial properties, mechanical, thermal, and chemical stability) of these membranes. This review focuses on applications of different types of nanomaterials: zero-dimensional (metal/metal oxide nanoparticles), one-dimensional (carbon nanotubes), two-dimensional (graphene and associated structures), and three-dimensional (zeolites and associated frameworks) nanomaterials combined with polymers towards novel polymeric nanocomposites for water and wastewater treatment applications. This review will show that combinations of nanomaterials and polymers impart enhanced features into the pristine membrane; however, the underlying issues associated with the modification processes and environmental impact of these membranes are less obvious. This review also highlights the utility of computational methods toward understanding the structural and functional properties of the membranes. Here, we highlight the fabrication methods, advantages, challenges, environmental impact, and future scope of these advanced polymeric nanocomposite membrane based systems for water and wastewater treatment applications.

## 1. Introduction

Water is essential for sustainable development, energy and food production, healthy ecosystems, and, of course, all life. Industrialization is at the core of urbanization and significantly contributes to the advancement of human welfare [1]. However, population growth, industrialization, and socio-economic growth have led to anthropomorphic climate change and pollution, and thereby the deterioration of water quality, especially in developing countries. One-third of the world’s population is suffering from scarcity of safe drinking water. This figure is expected to rise to two-thirds by 2025 [2,3]. Hence, one of the utmost global challenges is meeting the demand for safe drinking water [4].

Rapid industrial growth has exacerbated the production and widespread fouling of natural water resources [5]. These contaminants are of emerging concern because they are perceived as potential threats to human life and the environment [5,6]. Different classes of emerging contaminants, their source of origin, and their adverse health effects are listed in Table 1. Emerging contaminants can be primarily classified as organic, inorganic, microbial, perfluoroalkylated, and radioactive substances [3,6,7,8,9,10]. They do not degrade or hydrolyze easily and are persistent in the environment, resulting in bioaccumulation [6]. Even though the acute nature of any adverse health effects will depend on an individual’s susceptibility and the mode of contact with the body, the US EPA has established maximum concentration levels (MCLs) for these contaminants in drinking water. For example, heavy metals include cadmium with an MCL of 5 parts per billion (ppb), antimony with 6 ppb, lead with 15 ppb, and uranium MCL with 30 ppb [11,12,13]. Pesticides such as toxaphene and alachlor have an MCL of just 3 and 2 ppb, respectively [12], and many perfluoroalkylated compounds have MCL below 1 ppb, such as perfluorooctanoic acid and perfluorooctane sulfonate (both individually and combined) at less than 70 parts per trillion (ppt) [14]. The US EPA has recently announced that there is no safe level for the perfluoroalkylated compounds. Moreover, the lowest predicted no effect concentration (LPNEC) for a few contaminants in freshwater include 20 ppt for ciprofloxacin (antibiotic), 18 ppt for estrone (hormones) [9], and 560 ppt for 4 MBC (sunscreen) [15]. Given the potential health risks and low MCLs associated with these pervasive chemicals (shown in Table 1), there has been significant ongoing efforts to understand the occurrence and health consequences of these contaminants. This review will discuss many of the developing robust water purification technologies for the production of safe and clean drinking water. In addition to removing anthropomorphic contaminants, we will discuss various methods to remove minerals from seawater and brackish water. Desalination technologies that treat natural water resources have evolved tremendously in recent decades to support urban and industrial development in areas with limited water supply and/or high transportation or development costs [16].

Membrane technology has become indispensable in numerous industries such as food [24], pharmacy [25], textile [26], petroleum products [27], chemicals [28], lithium ion batteries [29], fuel cells [30], gas separation [31], and wastewater and drinking water treatment systems [32,33]. Compared to all other conventional water treatment methods, membrane technologies offer affordable solutions that support excellent contaminant rejection, low energy consumption, and easy availability of raw materials [34,35]. Over the past two decades, synthetic membranes have played an integral role in industrial and domestic applications, and have replaced commercially available cellulose-derived membranes (cellulose diacetate, cellulose triacetate, and regenerated cellulose) due to their high tolerance to stressful conditions [33,36,37]. Synthetic membranes can be fabricated using organic materials (polymers) or inorganic materials (metals, oxides, and ceramics). Membrane technology has the flexibility of employing a wide range of materials based on material type (ceramic: zirconia, titania, silica, alumina, etc.; metal: silver, palladium, copper, etc.; polymer: polyvinylidene difluoride (PVDF), polyether sulfone (PES), polysulfone (PSf), polyvinyl alcohol (PVA), polyetratfluoroethylene (PTFE), polypropylene (PP), polyamide (PA), polyimide, poly(1-vinylpyrrolidone) (PVP), polyvinyl chloride (PVC), polyacrylonitrile (PAN), etc.) and pressure driven membrane separation processes (microfiltration (pore size: 50–500 nm), ultrafiltration (pore size: 2–50 nm), nanofiltration (pore size ≤ 2 nm), reverse osmosis (pore size: 0.3–0.6 nm), and forward osmosis (pore size: 0.3–0.6 nm)) [3,32,35,37,38,39,40,41,42,43].

Inorganic and organic materials bring their own benefits and challenges in the development of synthetic membranes. Inorganic membranes have exceptional mechanical strength, high durability, and high tolerance to chemical oxidation or extremes of pH, but also have high manufacturing cost and little to no control on pore size distribution, which make them less likely to be suitable for industrial use [1,44,45]. Polymer (or organic) membranes are widely used technologies in water treatment due to their high degree of control over pore size distribution, high flexibility in operating conditions, ease of synthesis, and cost effectiveness [1,44]. These polymeric membranes are commercially available with differentiated porosities that can be tuned for applications such as suspended solids, oil emulsions and microbe removal (microfiltration (MF); for colloidal solids, viruses, humics, proteins/polysaccharides removal (ultrafiltration, UF); for heavy metals, dissolved organic matter, common pharmaceuticals or pesticides removal (nanofiltration, NF); for desalination and ultrapure water production (reverse osmosis, RO and forward osmosis (FO)) [37,46,47]. These targeted functional systems are fabricated as thin-film composite (TFC) membranes that have been widely used in membrane-based water purification systems [48]. TFC membranes are comprised of a non-woven fabric support layer on which a porous intermediate polymer (PES or PSf) layer (~50 nm) is combined with a thin, highly crosslinked dense PA layer (<200 nm) [35,49,50]. The top epidermal layer provides selectivity and/or separation while the porous substrate layer that is permeable to water and dissolved solute particles provides mechanical strength [32,51]. These membranes exhibit better salt rejection, higher water flux or permeability, and higher stability (chemical, mechanical and thermal) compared to commercially available cellulose-based membranes [50,51,52,53]. PSf and PES are the most commonly used materials for UF applications and are used as the standard base substrates for NF and RO composite membranes [32]. PP and PVDF are more commonly used for MF membranes [37,41]. However, there are key problems associated with TFC membranes. These membranes are prone to fouling, are highly hydrophobic, have low chlorine resistance, low mechanical strength, and demonstrate an inherent tradeoff between water flux and solute selectivity [4,33,35,44,54]. Apart from these, RO systems have a relatively high energy demand to desalinate feedwater [55,56]. Because these TFC have some inadequacies in achieving long-term viability and cost-effective membrane models, the incorporation of nanomaterials has emerged as an effective approach to overcome these application challenges.

**Figure 1:**
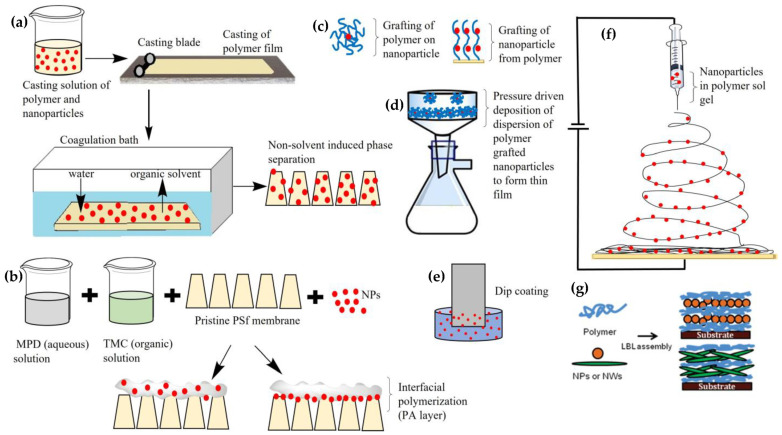
Various methods of integrating nanoparticles with polymer to form polymer nanocomposite membranes: (**a**) Schematic representation of one of the phase inversion methods (non-solvent-induced phase separation) for fabrication of polysulfone (PSf) layer. (**b**) Integration of nanoparticles either in the polyamide (PA) layer or as a thin layer at the bottom of the PA layer on top of PSf layer in nanocomposite membrane using interfacial polymerization method (MPD—m-phenylenediamine, TMC—trimesoyl chloride). (**c**) Short polymer strands grafted on a nanoparticle surface or nanoparticles grafted from the polymer membrane. (**d**) Pressure driven filtration of dispersion/solution of polymer and nanoparticles (polymer grafted nanoparticles example in this case). (**e**) Dip coating of polymeric membrane in a dispersion/solution containing nanoparticles. (**f**) Electrospinning of nanoparticles added in sol–gel (**g**). Layer-by-layer assembly of polymer and nanoparticles (NPs—nanoparticles, NWs—nanowires), [83], © American Chemical Society, 2008. For easy interpretation, spherical shapes are used for nanoparticles in most of the figures.

When nanomaterials are incorporated in the epidermal or porous intermediate layer or substrate/support, it produces polymer nanocomposite membranes. Compared to conventional TFC membranes, these nanocomposite membranes offer unique morphologies that overcome the limitations of pristine polymer membranes, leading to better performance and less energy demand. There are two ways of incorporating inorganic nanoparticles (NPs) into a polymer matrix. This can be either done by multilayer coating of NPs on polymer substrate or layer (thin-film nanocomposite membrane (TFNC)) [57,58] or dispersing NPs into a polymer blend, which forms into a cast (blended nanocomposite membranes) [39,59]. Blending can be achieved via phase inversion (PI) method, which is classified into four different types, and one of the common types used in fabrication is the non-solvent-induced phase separation (NIPS) method [39,60]. Fabrication of polymer membrane using NIPS has been shown in Figure 1. NPs are added to the solvent along with other additives and PSf (or any other polymer). This dope solution is casted on a glass plate with a casting blade set at a known gate height which is immediately transferred to a coagulation bath for polymer thin film to initiate the PI process. The membrane is peeled off the glass plate and kept in the bath to complete the PI process. By addition of hydrophilic fillers like NPs, there is a faster rate of organic solvent and non-solvent (water) exchange during the PI process, which leads to the diffusion of water from the water coagulation bath to polymer thin film, and the dissolution of walls between inner macrovoids and cavities leading to cavities of wider pores/voids and higher porosity (shown in Figure 1a) [61,62]. NPs can also be impregnated in the active epidermal layer (mainly PA) on the substrate during interfacial polymerization (IP) [63] or can be integrated as an intermediate layer between porous PSf substrate and semidense PA layer (shown in Figure 1b) [64]. This PA layer is prepared through the reaction between trimesoyl chloride (TMC) solution and m-phenylenediamine (MPD) solution during the IP process. NPs are added to either aqueous MPD or organic TMC phase depending on the hydrophilicity of NPs [65]. Multilayer coating of NPs on the substrate can be done by dip coating or layer-by-layer (LBL) deposition [66]. NPs loaded polymer sol–gel can also be electrospun at a high voltage into a nanofibrous membrane (shown in Figure 1f) [67,68,69]. There is the possibility of pressure driven membrane deposition of a dispersion of nanomaterials and the polymer [70,71]. Alternatively, NPs can be chemically cross linked to the polymer substrate [72,73], NPs can be grown in situ on the polymer surface [68], or the polymer can be covalently attached to the nanomaterials surface [74,75,76]. TFNCs are typically thin films of NPs coated on a polymer layer or substrate by dip coating, self-assembly, pressure-driven deposition, and other related techniques [39]. Mixed matrix membranes (MMMs) are membranes in which NPs are embedded as a dispersed phase into a polymer matrix, which can be achieved using techniques such as PI, electrospinning, crosslinking, LBL deposition, etc. [77,78,79,80,81,82]. This review will mostly focus on nanomaterials incorporated in the polymer substrate, but a few examples of other possibilities shall be discussed as well.

**Figure 2:**
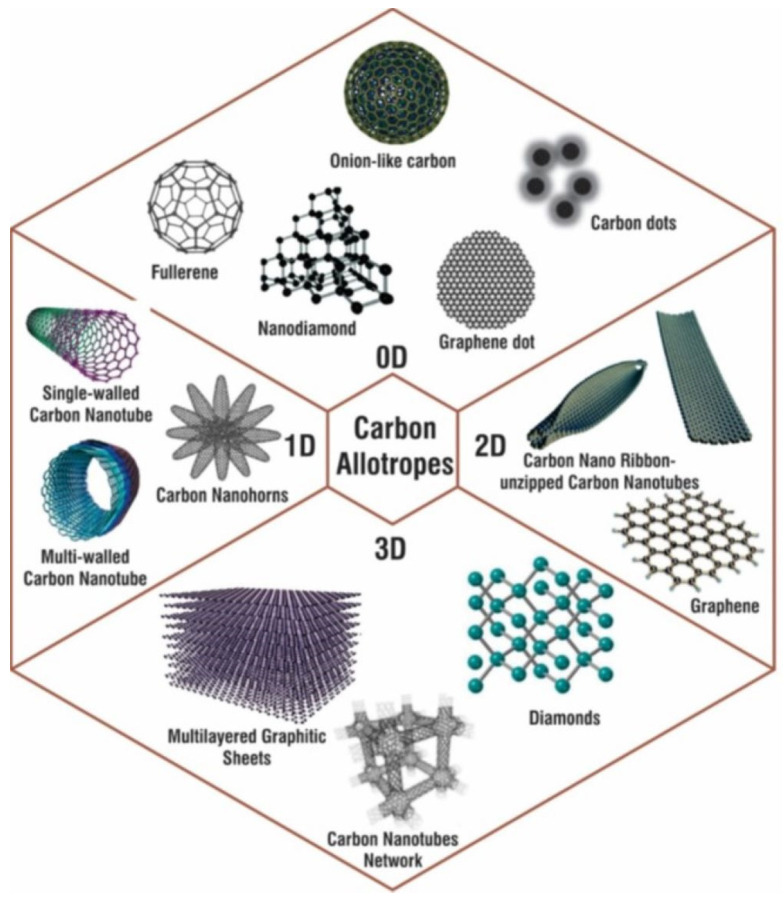
Classification of nanomaterials of carbon allotropes based on their dimensionality. Adapted with permission from Gaur et al. [85]. Copyright (2021) MPDI.

NPs differ from their larger bulk materials in that their size, shape, and dimensionality affect their properties and performance as a material. Specifically, when their size is reduced, the particles have extremely high specific surface area and surface-area-to-volume ratios. In a nanomaterial, at least one of the dimensions is in the nanoscale range of 1–100 nm. The nanomaterials are classified into zero-dimensional (0D), one-dimensional (1D), two-dimensional (2D), and three-dimensional (3D) nanomaterials. In 0D nanomaterials, all three dimensions are at the nanoscale. Examples include quantum dots, core shell NPs, nanospheres, etc. In 1D nanomaterials, two dimensions are at the nanoscale, giving the structures a rod like shape. Examples include nanowires, nanofibers, and nanotubes. In 2D nanomaterials, one dimension is at the nanoscale, giving the structures a sheet-like topology, e.g., graphene sheets. The 3D nanomaterials are not confined to the nanoscale in any dimension, which can include polycrystals, bundles of nanowires or nanotubes, and nanoporous solids. Examples include graphite, dendrimers, liposome, etc. [84]. For illustration, various dimensionalities of carbon allotropes are shown in Figure 2. The nanomaterial properties can be fine-tuned as desired by precisely controlling the size, shape, synthesis conditions, and necessary functionalization.

Additives like PVP and poly(ethylene glycol) (PEG) play important roles in membrane modification. They act as pore-forming agents and modify hydrophilicity and antifouling properties. However, dissolution or extrusion of homopolymer additives can lead to the deterioration of properties and the weakening of membrane performance. In this case, amphiphilic copolymers come to the rescue and show better compatibility, but these copolymers require costly and complex synthesis, making it difficult to achieve large scale production [39,86]. Maggay et al. investigated the amphiphilic nature of the copolymer of styrene and ethylene glycol methacrylate that was used to modify the PVDF membrane. It was found that the increase of the hydrophilic part led to the decline in anchoring sites, which led to a compromise in stability; the increase of the hydrophobic part led to the decrease of the antifouling property and increased protein adsorption on the surface. In addition to this, fine tuning of chain lengths of copolymer was required as well [87]. There are several reasons why there has been great interest in the development of polymeric nanocomposite membranes incorporating nanomaterials in drinking water and wastewater treatment systems. First, the incorporation of nanomaterials can implement extraordinary variations in polymeric nanocomposite properties such as permeability, selectivity, hydrophilicity, conductivity, magnetism, mechanical strength, thermal stability, and antimicrobial properties [35,41,44,88,89,90]. Second, there has always been a threat of NPs leaching out into the environment, whereas their incorporation into a hybrid polymer nanocomposite can mitigate the possibility of environmental discharge due to encapsulation [4,5,91]. Third, fouling in pristine polymeric membranes has been a serious problem. It is a well-known fact that foulants get adsorbed on the membrane surface due to van der Waals interactions, hydrogen bonding, and hydrophobic interactions [35]. Modification of the surface charge of polymeric membranes with hydrophilic components helps prevent or reduce undesirable foulant interactions and boost membrane longevity. For instance, modification of PVDF membranes has been performed by grafting or blending amphiphilic copolymers [92,93,94], introducing hydrophilic components [95,96,97], or by incorporating NPs in the PVDF substrate during PI fabrication methods [60,61,98]. Apart from this, antifouling properties can also be enhanced with NPs that introduce photocatalytic, self-cleaning, and photodegradable properties [35]. In addition, NPs with tunable porosities impart enhanced selective separation in these MMMs [99]. Thus, the addition of NPs has been beneficial for the long-term usage of polymer nanocomposites due to reduced membrane fouling. Because the fabrication of NPs often requires toxic chemicals, there has been ongoing research efforts in the implementation of sustainable methods to facilitate the widespread use of nanomaterials in water treatment [35].

It is important to have an optimum polymer/NP interphase/adhesion region to overcome agglomeration, which is one of the major challenges in the homogenous dispersion of NPs in a polymer blend. Agglomeration not only affects the performance and mechanical properties, but weak adhesion between the polymer and the agglomerated NPs can lead to composite failure due to the concentration of exerted force on weak spots [100,101,102]. Ashraf et al. showed that two grams of well dispersed and isolated 10 nm radius NPs can produce a remarkable interfacial area of 250 m^2^ within a polymer matrix [103]. When particles come in contact, they interact through van der Waals (vdW) attractive forces. The second interaction is electric double layer (EDL) repulsion, which arises due to the charged surface and surrounding counter ions and falls off exponentially with interparticle distance. Derjaguin–Landau–Verwey–Overbeek (DLVO) theory combines the vdW attractive force and the EDL repulsive force to understand the overall interactions between the NPs within the polymer matrix [104,105]. The other non-DLVO forces that influence aggregation are hydration forces and hydrophobic interactions. It is the interplay between these short-range thermodynamic interactions that determines the aggregation of colloidal particles. However, as the nanofiller concentration increases, there is a dominance of strong vdW forces that result in irreversible agglomeration [106]. External factors, such as the solvent removal process, add new forces such as capillary action that can promote NP aggregation as well [105]. Liu et al. performed molecular dynamics (MD) simulations and demonstrated that a homogeneous dispersion of nanofillers is achieved at the intermediate interphase interaction, which is contrary to conventional theories [107]. In this review, we have cited several filler-polymer combinations where an optimum concentration resulted in best interphase compatibility, properties, and performance, beyond which the performance deteriorated. Hence, it is crucial to manipulate the particles and minimize the colloidal agglomeration through methods such as mechanical agitation (like ultrasonication [108,109,110,111]), surface modification/functionalization to modify surface zeta potential [112], optimization of the incorporation procedure [113], etc.

**Figure 3:**
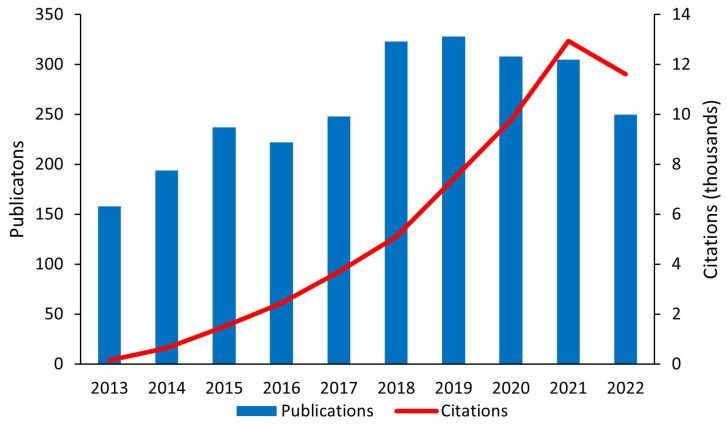
Research impact of thin-film polymeric nanocomposites analyzed using web of science database for the past decade.

TFC membranes are state-of-the-art and further progress has been made in their fabrication by introducing nanomaterials to formulate TFNC membranes [48]. TFNC membranes were first reported by Joeng et al. (2007), where the authors developed the first generation of MMMs by embedding NaA zeolite NPs in PA thin films interfacially polymerized on a PSf support. These RO membranes demonstrated a two-fold increase in water flux (9.37 → 16.96 ${L\;m}^{-2}h^{-1}$) and did not affect the solute rejection (93.9%, 2000 ppm feed concentration, 12.4 bar pressure), making them comparable to commercial RO membranes [114]. This paved the way for the exploration of nanomaterials to generate unique morphologies for preferential water flow and excellent salt rejection. Incorporation of NPs in TFNC contributes to the enhancement of membrane properties such as enhanced hydrophilicity, water permeability/flux, excellent salt rejection, removal of organic and inorganic contaminants, and enhanced resistance to chlorine and fouling [41,115,116,117,118]. TFNC polymeric membranes have drawn considerable attention in the past decade (as shown in Figure 3). This review focuses on the key ongoing advances in nanomaterial-modified polymeric thin-film membranes that—more specifically—benefit water and wastewater treatment technologies. 

We have further divided this review, focusing specifically on metal- or metal-oxide-based, carbon-nanostructures-based, zeolite-framework-based, and environmentally sustainable-materials-based polymer nanocomposite membranes. These nanocomposite membranes will be systematically evaluated for new properties and enhancement of existing properties that benefit from the introduction of NPs. The influence of different types of NPs, their concentration, their loading positions, their effect on morphologies, factors controlling the performance (hydrophilicity, antifouling, addition of surface charge, porosity, thermal, mechanical strength, change in surface roughness), and performances (permeability, selectivity/separation, rejection) will be comprehensively evaluated. This review details the delineation of updated findings and challenges associated with 0D, 1D, 2D, and 3D nanomaterials-based polymeric nanocomposites with a focus on MF, UF, NF, RO, and FO, which is beneficial to researchers for prospective materials and techniques. In addition to this, computational studies leading to better understandings of contaminant or foulant–membrane interactions, specifically in PA layer to examine antifouling behavior, are highlighted. Nanostructural forms of biopolymers are discussed to compare with synthetic inorganic nanomaterials. We will also discuss the environmental impact and future scope of these nanocomposite membranes. However, it should be noted that the membrane performance is highly dependent on the test conditions, which are different for various membrane applications (MF, NF, UF, RO, FO). Our efforts have primarily focused on a single type of NP-integrated membrane system, but have been extended to some combinatorial examples to demonstrate remarkable synergy between NPs when combined. Lastly, this review offers a diverse variety of polymer-nanoparticle thin-film combinations compared to previously published reviews, as shown in Figure 1. The aim of this manuscript is to offer a holistic overview of the extensive research conducted that can aid in the prospective selection of materials, combinations, and technologies primarily for water and wastewater treatment membrane solutions.

## 2. Incorporation of NPs in TFNCs/MMMs

### 2.1. Metal/Metal-Oxide-Based Nanocomposites

The incorporation of metal oxide NPs in polymers leads to the enhancement of various properties. For instance, enhancement in hydrophilicity is a common trait because these NPs can absorb hydroxyl groups and form a hydration layer on surface, imparting hydrophilicity in MMMs [119]. They have inherent antibacterial [108,120,121,122], antifouling [123,124,125,126], and magnetic properties [127,128,129,130]. These traits enhance water flux and rejection/adsorption capacity properties. Apart from this, low cost, photocatalytic degradation, self-cleaning, and low toxicity are other important features found in metal oxide NPs [33,119,131,132,133]. However, metal oxide NPs have issues with uncontrolled colloidal aggregation at higher concentrations due to organic–inorganic incompatibility [80,134,135], which affects the specific surface area and reactivity of these NPs and has a negative impact on the mechanical properties and performance [80] of these systems, resulting in increased viscosity and pore blocking phenomena [136]. The optimization of the fabrication procedure that allows for a homogeneous distribution of NPs within the polymer matrix and the targeting of an optimal threshold concentration should be considered to minimize agglomeration. There has been extensive work carried out proposing optimal concentrations of incorporated NPs to provide maximum water flux and the highest desalination/adsorption capacity in membranes incorporating metal oxide NPs [80,134,137,138,139,140]. Recently, Erdugan et al. fabricated PVC membranes with specially designed hexagonal platelets of ZnO which exhibited promising UF membrane performance without agglomeration issues [106]. Additionally, the risk of leached NPs present in the environment and potential toxic effects on human health is always an ongoing concern [141,142,143]. This incorporation must be tailored in a way that fits the best process tools for environmentally friendly, high-performance water treatment systems.

#### 2.1.1. Iron-Oxide-Based Nanocomposites

Iron is one of the most abundant and inexpensive elements present on earth and is widely used for geological and infrastructural purposes [144]. However, nano-dimensional Fe is highly reactive and unstable, and therefore the oxide forms have been extensively used [44]. This form of iron is used for incorporation in the polymer matrix as it is low cost, possesses hydrophilic properties, and has resulted in the enhancement of various properties discussed further in this section. Upadhyaya et al. evaluated protein permeability performance of hydrophobic and hydrophilic MMMs where hydrophilic and hydrophobic nanocomposite membranes consisted of casting solutions of polymeric spheres interconnected with quaternized poly(2-dimethylamino)ethyl methacrylate-coated iron oxide NPs and superparamagnetic NPs consisting of diblock copolymer and stabilizer-coated iron oxide NPs [127,145]. Application of a magnetic field in these membranes resulted in the enhancement of permeated flux and a reduction of protein fouling effects, allowing them to be used as antifouling nanocomposite membranes [146]. Kim et al. synthesized poly-N-phenylglycine nanofibers grafted onto reduced graphene oxide (GO) sheets intercalated with Fe_3_O_4_ NPs to form nanocomposites that exhibited a high degradation capacity of Cu(II) up to 95%. They used density functional theory (DFT) calculations and Perdew–Burke–Ernzerhof (RPBE) exchange–correlation functional to show that there is stronger binding due to deprotonated functional groups at higher pH compared to lower pH, resulting in high sorption efficacy [147]. To reduce agglomeration and enhance interphase compatibility, surfaces of NPs are modified as well. Nawi et al. fabricated the surface of Fe_3_O_4_ NPs with polydopamine, followed by functionalization with (3-aminopropyl)triethoxysilane (APTES) or chlorosulfonic acid, which were impregnated onto hollow PES fibers by the dry/wet spinning method. These nanocomposite membranes demonstrated an enhancement in water flux (82.60 → 137.23 ${L\;m}^{-2}h^{-1}{\mathrm{bar}}^{-1}$) and adsorption capacity (71.92 → 92.16%) of bovine serum albumin (BSA) when compared to pristine PES fiber [148].

#### 2.1.2. Silver/Zinc-Based Nanocomposites

Foulant build-up can lead to a decrease in membrane water flux with increased run time, increasing operational costs and shortening membrane life [149,150]. Membrane fouling can be of various types ranging from crystalline scaling, organic fouling, microbial fouling, or particulate and colloidal fouling [151,152]. These occur due to the interaction of the membrane with different sources. Biofouling has detrimental effects on membrane systems and accounts for 45% of membrane fouling [151]. The incorporation of NPs into membranes can offer affordable solutions for long-lasting sustainable membranes by increasing the resistance to membrane biofouling. Ag NPs are well known antibacterial agents. The mechanism of antibacterial activity of Ag stems from the denaturation effect of Ag ions, which causes the condensed DNA to lose its replication ability. Ag ions interact with thiol groups present in amino acids, resulting in the inactivation of bacterial proteins [153]. Ag ions have an affinity for sulfhydryl groups exposed on bacteria or viruses, disrupting the H_2_ energy transfer system in microorganisms due to the sulfur and Ag bond [154]. Khare et al. added PEG-soaked Ag grown activated carbon microfibers and nanofibers (Ag-ACFs/CNFs) in situ during emulsion polymerization of PVA, which was casted into a film followed by the creation of laser ablation microchannels to expose Ag-ACFs/CNFs dispersed within the polymer matrix. This metal–carbon–polymer-nanocomposite-based contractor inhibited the growth of gram-negative *Escherichia coli* (*E. coli* K-12) and gram-positive *Staphylococcus aureus* (*S. aureus* RN4220) bacterial strains instantaneously under flow conditions due to the antibacterial property imparted from Ag NPs [155]. Besides acting as a scaffolding support to Ag NPs, CNFs enhanced the tensile strength and thermal stability of the film. There are also reports on the antibacterial property of ZnO in the literature. From previous works, it is well known that the antibacterial activity of ZnO is related to the generation of H_2_O_2_ on the surface [156,157]. Jo et al. modified PES membranes with PVP-grafted and poly(1-vinylpyrrolidone-co-acrylonitrile) (P(VP-AN))-grafted ZnO NPs by the NIPS process. PVP and P(VP-AN) imparted hydrophilicity while ZnO imparted antibacterial properties. Antibacterial activity was measured in accordance with the JIS Z-2801 standard and 0.5 wt.% loaded ZnO-modified membranes showed an enhanced antibacterial activity (0.2 → 6.1) toward *E. coli* (ATCC 8739) and *S. aureus* (ATCC 6538P) when compared to pristine membranes. These PES/polymer-grafted ZnO membranes demonstrated an increase in water flux and hydrophilicity, but a slight decrease in PEG rejection with an increase in filler content (>4 wt.%); there were improved antifouling characteristics compared to the PES membrane only, with no ZnO leaching observed in the modified membrane [158]. Mousa et al. electrospun 0.2 wt.% loaded ZnO NPs in a blend of PSf and cellulose acetate and coated with a 0.1 M NaOH solution to fabricate a superhydrophilic nanofibrous membrane. This membrane showed a decrease in water contact angle (WCA) (72.86 → 13.17°), comparable tensile strength, enhanced water flux (20 → 460 ${L\;m}^{-2}h^{-1}$), strong antibacterial activity against *E. coli* with a bacterial growth inhibition zone diameter of 10 ± 0.6 mm, but low flexibility [108]. Hong and He incorporated a PVDF membrane with ZnO NPs and the results revealed an improvement in photocatalytic self-cleaning efficiency (62 → 93%) and water flux (66.6 → 147.2 ${L\;m}^{-2}h^{-1}$), and a decrease in WCA (63.21°) with an increase in ZnO NPs content, but led to a decrease in mechanical strength and chemical oxygen demand removal efficiency after loading was exceeded beyond 0.01 wt.% [159].

#### 2.1.3. Silica-Based Nanocomposites

**Figure 4:**
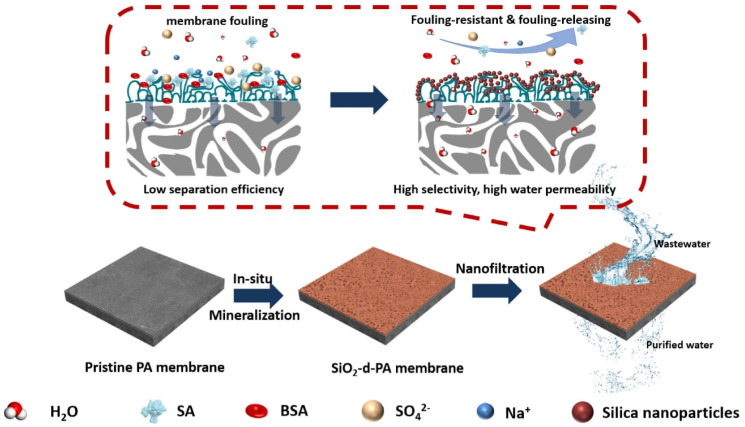
Fabrication of SiO_2_−coated polyamide-based membrane for high-capacity rejection and antifouling activity. Adapted with permission from Istirokhatun et al. [123]. Copyright (2021). Elsevier.

The incorporation of silica NPs has been a widely used method due to their chemical, structural, and thermal stability, facile suspension in aqueous solution, and environmentally benign property [160,161,162]. For instance, when silica NPs were incorporated in PVDF membranes, the nanocomposite membranes exhibited higher thermal stability, hydrophilicity, and improved levels of selectivity due to the presence of silica NPs [163]. Additionally, mesoporous silica are porous nanostructures incorporated to introduce a uniform pore size distribution [164]. Silica NPs have been incorporated within the polymer matrix or anchored on the surfaces of electrospun polymeric fibrous membranes. Pi et al. impregnated silica NPs on electrospun poly(vinylidene fluoride-hexafluoropropylene) (PVDF-HFP) to form superhydrophilic multistructured nanofibrous membranes for the removal of Cu(II) ion, which resulted in an adsorption capacity of 21.9 ${\mathrm{mg}\;g}^{-1}$ [165]. A Freundlich adsorption isotherm and a pseudo-first-order kinetic model was best fit for the experimental data. Teng et al. electrospun PVP combined with SiO_2_ to form mesoporous fiber membranes with thioether group functionalization for selective adsorption of Hg(II). These membranes demonstrated an adsorption capacity of 852 ${\mathrm{mg}\;g}^{-1}$ with an 18% molar concentration of organosilane precursor [166]. Keshtkar et al. obtained nanofibrous membranes by electrospinning a sol–gel of dispersed SiO_2_ NPs (7–25 nm particle size), PVP, APTES, and tetraethylorthosilicate, and demonstrated removal of Cd(II), Pb(II), and Ni(II) with an adsorption capacity of 157.4, 158.3, and 63.0 ${\mathrm{mg}\;g}^{-1}$, respectively. These experiments were conducted with initial concentrations of these heavy metal ions ranging between 30 and 500 ${\mathrm{mg}\;L}^{-1}$ at pH 6. The BET surface area of these nanoporous membranes was 65.647 $m^{2}g^{-1}$ and the adsorption isotherm best correlated with a Langmuir model. The activity coefficient of the adsorbate was less than 8 ${\mathrm{kJ}\;\mathrm{mol}}^{-1}$, indicating that the adsorption was a physical process [167]. Istirokhatun et al. demonstrated antifouling activity in the SiO_2_-coated PA-based membrane owing to the hydration property of SiO_2_. The fabrication of this membrane is demonstrated in Figure 4 [123]. A summary of key enhancements in membrane properties due to the inclusion of different types of SiO_2_ nanoclusters has been illustrated in Table 2.

**Figure 5:**
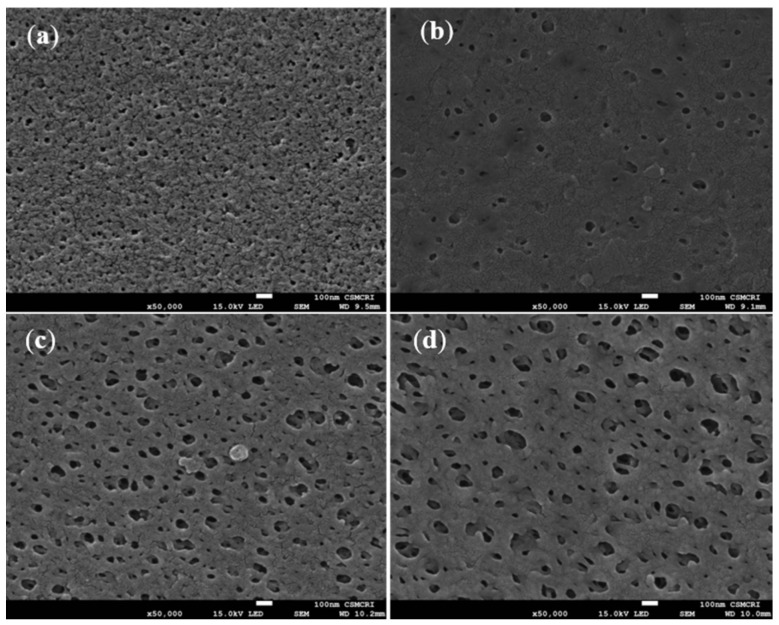
Scanning electron microscopy (SEM) revealed that pore size is directly proportional to diatom loading. SEM surface porosity images of (**a**) Polysulfone (PSf) with 0.0% of diatom, (**b**) PSf with 0.1 wt.% of diatom, (**c**) PSf with 0.2 wt.% of diatom, (**d**) PSf with 0.5 wt.% diatom (×50,000, 100 nm scale bar in all micrographs). Adapted from Paidi et al. [172]. Copyright (2022) MDPI.

Taking the toxicity of NPs into consideration, Paidi et al. demonstrated the application of 3D mesoporous silica derived from marine diatom *T. lunidiana* cultures impregnated in PSf membranes. These membranes exhibited enhanced hydrophilicity and had uniformly distributed large pores and low surface roughness as observed by scanning electron microscopy (SEM) (shown in Figure 5) and atomic force microscopy. Silica frustules extracted from biomass were cleaned using corrosive nitric acid. In addition to the selection of biomass-derived products, the processing of these materials should also be considered in regard to the environmental impact. The highest loaded PSf nanocomposites (0.5% diatom) demonstrated a water flux of ~807 ${L\;m}^{-2}h^{-1}{\mathrm{bar}}^{-1}$ at 20 psi operating pressure and a removal rate of 98.5% and 94.8% for 500 ${\mathrm{mg}\;L}^{-1}$ of BSA and 0.1 M of rhodamine, respectively [172].

#### 2.1.4. Titania-Based Nanocomposites

In addition to antifouling properties, TiO_2_ NPs also possess tunable morphologies, facile surface functionalization/modification, chemical stability, low costs, self-cleaning properties, and photocatalytic activity for organic contaminants, making them suitable for membrane technology [173,174,175,176,177,178]. It is already known that photocatalytic properties of TiO_2_ NPs help in the degradation of water contaminants in the presence of an energy source [179,180,181,182,183,184]. The mechanism of degradation lies in the absorption of energies higher than the semiconductor band gap, resulting in the excitation of electrons from the valence band to the conduction band. This leaves a hole in the valence band that can react with water molecules to generate highly reactive hydroxyl radicals that can oxidize organic contaminants [132]. Aoudjit et al. immobilized Ag-functionalized TiO_2_ NPs into the poly(vinylidene fluoride-hexafluoropropylene) matrix and tested photocatalytic activity against an emerging contaminant, metronidazole, under solar radiation. The results revealed a maximum degradation efficiency of 100% with an initial metronidazole concentration of 10 ${\mathrm{mg}\;L}^{-1}$ at a pH of 7 under 5 h of solar radiation [185]. Zhang et al. prepared a PVDF nanocomposite membrane by pre-dispersing TiO_2_ NPs via PEG additive, which ensured uniformly spaced surface pores, larger porosity, high water flux, negative zeta potential, and an increased hydrophilicity of the membrane. This membrane exhibited a higher interaction energy peak compared to the control membranes, which signified the increasing difficulty with which foulants interact with or attach onto membrane surfaces when evaluated using extended DLVO theory. This, along with low flux decline from the filtration experiment, corroborates the enhanced antifouling performance [186]. Like other metal-based NPs, TiO_2_ NPs have been extensively used in MF [187,188,189,190], NF [191,192,193,194,195], UF [196,197,198,199,200], and RO [201,202,203] applications. Yu et al. incorporated TiO_2_ NPs in a dope containing 18 wt.% PVDF and 5 wt.% additive PVP to formulate hollow-fiber UF membranes using the sol–gel method and the blending method. Compared to a pristine PVDF membrane, the addition of 1 wt.% loaded TiO_2_ NPs using the sol–gel method enhanced the hydrophilicity (lowering of WCA, 79.13 → 34.91°), thermal stability, mechanical strength (1.71 → 2.26 MPa), and water permeation (~110 → 244 ${L\;m}^{-2}h^{-1}$) of this UF membrane, resulting in improved antifouling properties. The hydrophilicity and permeability subsequently decreased beyond 1 wt.% loading due to an increase in viscosity and pore blocking phenomena. The sol–gel method resulted in uniformly dispersed TiO_2_ particles in the polymer matrix compared to the blending method [198]. Key enhancements, as well as the loading % at which TiO_2_ agglomeration occurred, have been highlighted in Table 3 to consolidate the threshold concentration values used by researchers to obtain best performance.

**Table 3:** A summary of key enhancements in properties of TiO_2_ nanoparticle-based nanocomposite membranes.

Membrane Type	Enhancement and Agglomeration Due to Modification	Reference
PSf UF membrane with PANI-coated TiO_2_ NPs and PEG as additives fabrication by PI process	Enhanced porosity, permeability, hydrophilicity, water uptake, antifouling property with a rejection of 68% and 53.78% for Pb^2+^ and Cd^2+^, respectively. Agglomeration @ 1.5 wt.% loading.	[204]
PSf-based PANI-coated TiO_2_ NPs-coated PA nanocomposite hollow fiber membrane	Enhanced hydrophilicity and antifouling property with a rejection of 81.5% and 96.5% for Reactive Black 5 and Reactive Orange 16. Agglomeration @ 1 wt.% loading.	[125]
TiO_2_ NPs incorporated into PSf UF membrane	Better porosity, hydrophilicity, and antifouling property. Tiny aggregates @ 2.0 wt.% loading	[124]
Addition of TiO_2_ NPs in a PVDF and sulfonated PES blend membrane fabrication by PI method	Enhanced hydrophilicity, antifouling, photo-bactericidal effect against *E. coli*, higher FRR (86.2%). NPs loading negative effect on pure water flux. Agglomeration ≥ 4 wt.%.	[197]
Addition of TiO_2_ NPs in microporous PES membrane	Enhanced hydrophilicity, mean pore size and permeation property, flux (3711 ${L\;m}^{-2}h^{-1}$), mechanical strength, thermal stability. Agglomeration @ 4–5 wt.% loading.	[110]
Electrospun nanofibers from a blend of PVP, PVDF and TiO_2_ NPs (oil–water separation)	Enhanced hydrophilicity, mechanical strength, chemical stability, and antifouling property with high separation efficiency (98.4%) and FRR (95.68%) (Schematic shown in Figure 6)	[205]
PSf membrane using TiO_2_ nanorods forming flower-like structures used as additive	Enhanced hydrophilicity, high surface area, self-cleaning efficiency (68.8%), antifouling activity	[111]
L-cysteine-surface-modified TiO_2_ NPs incorporated in PES membrane by PI process	Enhanced water flux, direct red-16 (98%) and liquorice (90%) removal, hydrophilicity, antifouling. Agglomeration @ 1 wt.% loading	[140]

PANI: polyacrylonitrile; PSf: polysulfone; PEG: poly(ethylene) glycol; PI: Phase inversion; PVP: poly(1-vinylpyrrolidone); PVDF: polyvinylidene difluoride; UF: ultrafiltration; FRR: flux recovery rate.

Hosseini et al. fabricated UF membranes by incorporating TiO_2_ NPs and observed that a 7 wt.% loading of TiO_2_ NPs resulted in the optimal properties of higher porosity, higher hydrophilicity, water flux, lower flux decline, mechanical stability, high oil rejection, and antifouling properties. However, higher loading (10 wt.%) resulted in a non-uniform dispersion, aggregation, and pore blocking of the membrane, which in turn resulted in a defective structure and a reduction in mechanical strength. Hosseini et al. tested the stability of the matrix and observed a 10 wt.% loading to be leaching from the polymer matrix, which is possible due to non-covalent binding of NPs onto the membrane surface [196]. This is noteworthy while addressing the concern of NPs leaching into the environment. Mahdhi et al. used the Lifshitz and Young–Laplace theories’ analysis to show that the incorporation of threshold volume fraction of TiO_2_ NPs in PVDF, chitosan (CS), and cellulose acetate led to the conversion of the hydrophobic matrix to hydrophilic in nature, which resulted in the draining of water inside the nanopores without the need for external pressure or energy opening new avenues for green and sustainable NF membranes [191].

**Figure 6:**
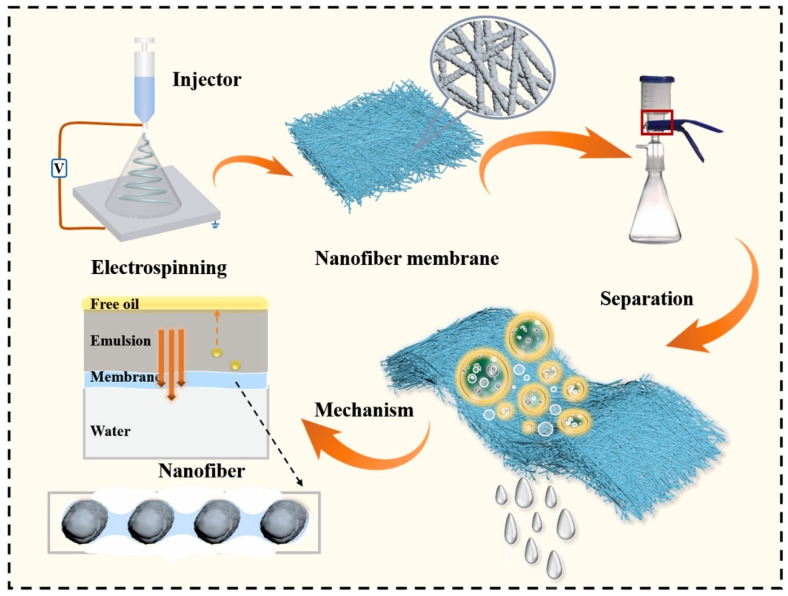
Fabrication of blend containing polyvinylidene difluoride and TiO_2_ nanoparticles into a membrane for oil–water separation. Adapted with permission from Du et al. [205]. Copyright (2021) Elsevier.

### 2.2. Carbon-Nanostructure-Based Nanocomposites

Numerous studies on carbon-based nanomaterial adsorbates have been conducted because these materials exhibit high specific surface area, mechanical strength, uniform porosity, thermal stability, surface reactivity, and chemical stability to harsh conditions [206,207,208,209,210]. The performance of carbon nanostructures is superior in terms of high-water flux, high ion rejection, and antifouling properties. However, it is critical to note that carbon nanostructures have intrinsically poor dispersibility and thus agglomerate, which can be avoided by surface functionalization. Carbon nanostructures have been specifically used as nanofillers to enhance the mechanical strength and viscoelasticity of polymer matrices [211,212,213]. Both carbon nanostructures and polymer membranes can adsorb or capture contaminants; however, their mechanism will be different based on the structure, morphology, stacking arrangement, and presence of surface functional groups. Additionally, this section will provide a comprehensive summary of computational studies used to understand foulant–membrane interactions and antifouling phenomena in PA membranes.

#### 2.2.1. Carbon-Nanotube-Based Nanocomposites

This class of materials has attracted widespread interest in the scientific community for a wide range of applications due to their unique properties. The inner diameter of CNTs can be adjusted within a narrow range to ensure high-efficiency performance of CNT-based membranes [214]. Compared to NF and RO membranes, CNT-based membranes are more resistant to biofouling, thus reducing operating costs [214]. Incorporation of CNTs in the polymer matrices leads to better thermal, mechanical, electrical, and rheological properties, even at low concentrations [213,215,216]. However, CNTs usually aggregate together because of van der Waals interactions, thus the efficient fabrication of these matrices is critical in order to optimize the performance of these nanocomposites [217]. Shawky et al. synthesized nanocomposite membranes by grafting a PA substrate with multiwalled carbon nanotubes (MWCNTs). It was found that a loading of 15 ${\mathrm{mg}\;g}^{-1}$ of MWCNT resulted in an increase in salt rejection (24 → 76%) and mechanical properties (34.3 Mpa) with only a small decrease in water flux (32 → 28 ${L\;m}^{-2}h^{-1}$) [218]. Dumee et al. fabricated a dense layer of PA on a support substrate of a hydroxyl-functionalized CNT mesh, which resulted in higher porosity (>90%), low WCA (<20°), and high water uptake capacity (17 wt.%) compared to PSf membranes paving the way for FO and RO applications [219].

**Figure 7:**
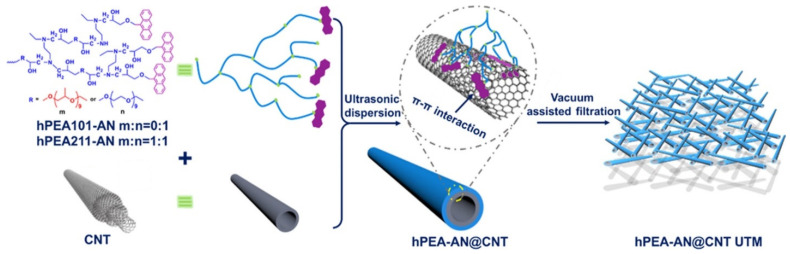
Schematic of the fabrication of anthracene-ending hyperbranched poly(ether amine)-coated carbon nanotube thin films formed by vacuum filtration. Adapted with permission from Zhang et al. [221]. Copyright (2016) American Chemical Society.

Lee et al. prepared a microporous membrane by incorporating 0.5 wt.% CNT in PSf support matrix and demonstrated an increase in water flux (268 → 342 ${L\;m}^{-2}h^{-1}{\mathrm{bar}}^{-1}$), surface porosity (1.4 → 3.8%), and salt rejection (97.4 → 97.7%) while maintaining the WCA (66.2 → 66.6°). This paved the way for designing optimized supports for FO and pressure-retarded osmosis [220]. Using π-π stacking and hydrophobic interactions, Zhang et al. designed an ultrathin film (1.5 µm) with an entangled mesh of CNTs uniformly coated with hyperbranched anthracene ending poly(ether) moieties (schematic is shown in Figure 7) [221]. These membranes displayed selective adsorption towards dyes, separation efficiency of up to 100% for molecules with similar backbones and the same charge states, and the ability to be regenerated without compromising efficiency. For dyes such as erythrosine B and Evans blue, adsorption capacities went up to 300 ${µ\mathrm{mol}\;g}^{-1}$ for an initial concentration of 300 ${µ\mathrm{mol}\;L}^{-1}$ in 6 mL phosphate-buffered aqueous media at pH 7.2, proving these nanocomposites to be promising nanoadsorbents for wastewater treatment [221]. Wu et al. fabricated an electrospun porous support layer of CS/PVP/PVA and single-walled CNTs (SWCNTs) were incorporated by the electrospraying technique along with CS and PVP. Optimized UF thin-film membranes exhibited a water flux of 1533.26 ${L\;m}^{-2}h^{-1\;}$, which is comparable to commercial PVDF UF membranes. These membranes also achieved excellent dye rejection (malachite green (MG), 87.20%; MB, 76.33%; CV, 63.39%) and heavy metal removal (Cu^2+^, 95.68%; Ni^2+^, 93.86%; Cd^2+^, 88.52%; Pb^2+^, 80.41%), in addition to having enhanced antifouling properties [217].

#### 2.2.2. Graphene/Graphene Oxide (GO)/Reduced Graphene Oxide (rGO)-Based Nanocomposites

**Figure 8:**
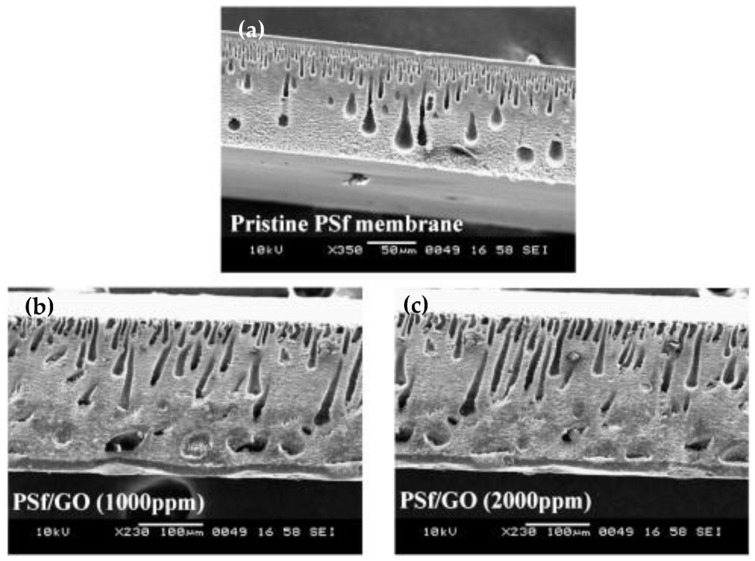
Cross sectional SEM illustration of (**a**) pristine PSf, (**b**) 1000 ppm GO loaded PSf, (**c**) 2000 ppm GO loaded PSf. Adapted with permission from Ganesh et al. [232]. Copyright (2013) Elsevier.

Due to the hydrophobicity and mono-atomic thickness, graphene has been extensively researched for membrane separations. Water permeation is extremely restricted in graphene due to the blocking of passage by the delocalized electron clouds due to *π-π* conjugation in the aromatic rings in graphene [222]. However, the adjacent interlayer distance can be effectively enlarged via intercalation, such as oxidation and subsequent exfoliation, or defects can be created in the form of pores to enhance membrane permeability while maintaining salt rejection [223,224]. GO contains several oxygen-rich functional groups (carboxylic acid, hydroxyl and epoxide groups), making this lamellar 2D structure hydrophilic, easily compliable to surface modifications, exhibit better permeability, and have good dispersibility in a variety of solvents [222,225] Due to this oxidation, GO becomes insulative, having poor thermal conductivity compared to graphene. Hydrophobicity, electrical, and thermal conductivity is partially restored by reducing GO as rGO [226]. Each of these forms of graphene is unique and has been extensively investigated for water and wastewater treatment. Zhang et al. prepared a nanofibrous membrane by performing an imidization reaction on electrospun poly(amide) acid and directly depositing rGO on the polyimide membrane by dip coating along with solvothermal reduction of GO. This membrane achieved a 99.19% oil–water separation efficiency, water flux up to 2040.04 ${L\;m}^{2}h^{-1},$ and retained its mechanical integrity under harsh conditions making it an attractive candidate for wastewater treatment [227]. Najafabadi et al. obtained a nanofibrous membrane by electrospinning CS along with GO. SEM analysis of GO loaded up to 0.7% found a decreased diameter of the fibers because of the electrical effect GO had on the precursor gel used for electrospinning. Analysis of the adsorption kinetics was consistent with external as well as internal diffusion during the sorption process, resulting in a heavy metal ion adsorption capacity of 423.8, 461.3, and 310.4 ${\mathrm{mg}\;g}^{-1}$ for Pb^2+^, Cu^2+^, and Cr^6+^, respectively at 45 °C with an equilibrium time of 30 min [228,229]. Kim et al. described the creation of a FO composite membrane by entwining GO sheets with a cross linked poly(Nisopropylacrylamide-co-N,N’-methylene-bisacrylamide) network on a highly porous nylon substrate. This membrane was less than 40 nm and demonstrated a water flux of 25.8 ${L\;m}^{-2}h^{-1}$ and a salt rejection of 99.9%. The membrane had excellent chlorine resistance along with structural stability and the potential to be utilized for FO [230]. Wang et al. hot-pressed rGO on an electrospun PAN membrane to obtain a NF desalination membrane. It exhibited a higher water flux of (8.41 → 15.0 ${L\;m}^{-2}h^{-1}$) compared to the pristine PAN membranes measured at 10 bars. Moreover, 81% of the initial flux was regained after regeneration. Due to the size exclusion effect, separation rejections of 90.0% and 23.8% were achieved for MgSO_4_ and NaCl, respectively, where the rGO nanochannels were considered to be narrower than the hydrated ion size of SO_4_^2−^, but wider than the Cl^−^ hydrated ion size [231]. Ganesh et al. incorporated GO into PSf membranes using the wet PI method, resulting in an introduction of macrovoids (shown in Figure 8), enhanced hydrophilicity, water flux, and Na_2_SO_4_ rejection (>40 → 72%) at 4 bar pressure using 2000 ppm loading [232].

Table 4 lists the benefits of impregnating GO in polymer substrates while categorizing different filtration studies to help rationalize the choice of MF, UF, NF, or RO so as to maximize either selectivity or permeability. Typically, the water flux decreases from MF towards RO as the pore size decreases. These membranes are capable of a wide variety of applications ranging from filtration, separation, adsorption, rejection, antifouling, and self-cleaning, among others, as cited with specific examples below [233].

**Table 4:** A summary of membrane types, applications, and enhancements in properties of nanocomposites due to incorporation of GO.

Membrane	Application	Results (Compared to TFCs)	References
rGO/PVDF	MF (1 bar)	Enhanced water flux: 1024 ${L\;m}^{-2}h^{-1}$; Acetaminophen rejection: 72%; Triclosan rejection: 81%; Enhanced antifouling	[234]
TiO_2_/GO/PVDF	UF (1 bar)	Water flux: 487.8 ${L\;m}^{-2}h^{-1}$; BSA rejection: 92.5%; Enhanced photodegradation efficiency; Enhanced antifouling; Self-cleaning	[235]
TiO_2_@GO/PES	UF (1 bar)	Water flux: 109.8 ${L\;m}^{-2}h^{-1}$; BSA rejection: 99.1%; MB photodegradation rate: 95.1%; FFR: 86.1%	[236]
Ag@GO/PVDF	UF	Water flux: 491 ${L\;m}^{-2}h^{-1}$; Flux loss: 21%; Improved hydrophilicity (86.1 → 62.5°); mechanical strength (1.94 → 2.13 MPa); Enhanced antifouling due to GO	[237]
GO-ND/PVC	UF (2 bar)	Improved Water flux (0 → 0.15 wt.%): 200 → 440 ${L\;m}^{-2}h^{-1}$; BSA rejection: 95.08%; Flux recovery: 83.07%; Enhanced hydrophilic, antifouling, and mechanical strength	[238]
GO/PANI/PVDF	NF (1 bar)	Enhanced water flux (0 → 0.1% wt./v GO): 112 → 454 ${L\;m}^{-2}h^{-1}$; BSA rejection: 38.6 → 78.3%; Allura red: ~80 → 98%; Methyl orange: ~80 → 95%; Enhanced hyrophobicity; degradation temperature: 398 → 470 °C; Improved Tensile strength: 32 → 90 MPa, Enhanced antifouling	[239]
COOH-GO/PA	NF (10 bar)	Enhanced water flux (0 → 0.07% wt./v GO): 110.4 ${L\;m}^{-2}h^{-1}$; New Coccine (dye) rejection: 95.1%; NaCl rejection: 25%; Improved hydrophilicity and surface charge density	[240]
GO/PPS	NF (0.3 bar)	Enhanced flux: 325.65 ${L\;m}^{-2}h^{-1}$; Methyl blue rejection: ≥99%; Methylene blue rejection: ~99%; Rhodamine B (RhB) rejection: >99%	[241]
rGO-NH_2_/PA	NF (2 bar)	Enhanced water flux (0 → 50 ${\mathrm{mg}\;L}^{-1}$ rGO-NH_2_): 30.44 → 38.57 ${L\;m}^{-2}h^{-1}$; Salt rejection: NaCl: 26.9%, Na_2_SO_4_: 98.5%, MgSO_4_: 98.1%, CaCl_2_: 96.1%; Improved antifouling properties	[242]
Zeolite/GO/PVDF	RO (55 bar)	Enhanced water flux (GO:Zeolite: 0.07): 15.6→ 34.5 ${L\;m}^{-2}h^{-1}$; Enhanced salt rejection: 82.8 → 96.86%; Higher porosity; Improved hydrophilicity	[243]
GO/PSf	RO (55 bar)	Enhanced water flux (0 → 0.5 wt.% GO): 27.2 → 35.6 ${L\;m}^{-2}h^{-1}$; NaCl rejection: 98.8 → 99.2%; Higher porosity: 63 → 71.1%; Surface free energy: −91.63 → −108.68 ${\mathrm{mJ}\;m}^{-2}$ (higher wettability); Enhanced tensile strength: 17.2 → 23.6 MPa	[244]

GO: graphene oxide; rGO/PVDF: Injection of rGO dispersion into PVDF membrane; TiO_2_/GO/PVDF: Blending of PVDF with TiO_2_ and GO; TiO_2_@GO/PES: layer-by-layer self-assembly of TiO_2_-loaded GO as few layers on PES membrane; Ag@GO/PVDF: Blending of PVDF with Ag-loaded GO; GO-ND/PVC: Incorporation of GO grafted with nanodiamond-COOH (GO-ND) into PVC membrane via IP method; GO/PANI/PVDF: Incorporation of GO and PANI in PVDF (PI); COOH-GO/PA: Incorporation of Carboxyl functionalized GO in polyamide (PA) membrane impregnated on PSf substrate via IP; GO/PPS: Nanographene GO stacked on the surface and pore channels of poly(p-phenylene sulfide) membrane by crosslinking through Ca^2+^, Cu^2+^, and Mg^2+^ (solution casting); Embedding amino rGO (rGO-NH_2_) into PA layer on the inner PES hollow membrane (IP process): rGO-NH_2_/PA; zeolite/GO/PVDF: Incorporation of zeolite and GO in PVDF membrane (solvothermal method); GO/PSf: Thin film of PA on Incorporation of GO in PSf (PI method).

#### 2.2.3. Computational Studies

MD and DFT calculations have been extensively used in water purification and treatment systems [245,246,247,248]. Simulation studies of nanostructured carbon-based thin films have gained increased effort by researchers, offering new possibilities and understandings of the fundamental structural and functional properties of these films. Despite modest success, the directional alignment of incorporated CNTs as nanofillers within the polymer matrix remains a matter of significant effort. Successfully aligned CNT morphology can yield maximum theoretical values of salt rejection and water flux. The focus of subsequent research has addressed this issue using computational studies. Briefly, Yang et al. annealed vertically aligned (VA) CNT arrays at 1500 °C, etched to open the CNTs and embedded within a spin-coated thin layer of polydimethylsiloxane (PDMS) to form a VA open ended hybrid membrane. The transport mechanism of various classes of molecules (C_6_H_5_OH, CO_2_, and N_2_) was evaluated using DFT calculations. It was found that the adsorption energy of phenol on the PDMS chain, the external wall, and the internal wall of CNT were −81.0, −69.5, and −208.6 ${\mathrm{kJ}\;\mathrm{mol}}^{-1}$, respectively, using DFT calculations. From experimental results, they found that the single gas permeability of CO_2_ and N_2_ and binary gas (CO_2_/N_2_) separation had superior performances compared to unannealed, close-ended, and unaligned control membranes [249]. Bisignano et al. created an ab initio methodology to study the high-capacity rejection of the small molecular weight emerging contaminant racfluoxetine, glucose, and other small molecules (ethanol, glucose, water) without compromising the membrane flux. This was done by simulating VA MWCNT arrays embedded within polyester or CS films to form a nanocomposite membrane. This novel algorithm included a study of functionalized MWCNT edge atoms at their open ends. The ends of the tubes were functionalized with polymers with intrinsic microporosity PIM-1 monomers. It was observed that the rejection of molecules was in accordance with a size exclusion mechanism with the highest rejection of racfluoxetine achieved using MWCNTs with 4.44 nm internal diameters. Aligning the MWCNTs can produce a flux that is three times higher than the unfunctionalized membrane. MWCNT with 4.44 nm diameter provided the best tradeoff between water permeability, effective width for maximum functionalization, and density of VA tubes [250].

In a study by Kim et al., a nanocomposite membrane was synthesized using intercalation of rGO functionalized with deprotonated poly-N-phenylglycine and Fe_3_O_4_ NPs that exhibited 95% degradation capacity of Cu(II). DFT calculations with RPBE exchange–correlation functionals were used to predict sorption affinity for Cu(II) at low and high pH. They found that there is stronger binding at high pH compared to low pH due to deprotonated functional groups. The adsorption process is best characterized as chemisorption at high pH but physisorption at low pH [147]. Khajouei et al. fabricated an antifouling UF membrane by incorporating GO NPs in PSf polymer matrix and used OPEN-MX cluster computing to evaluate the optimal loading percentage for GO deposition within a range (0.25, 0.5, 0.75, and 1 wt.%). They found that a loading of 0.75 wt.% was the most optimally stable state and, experimentally, this loading resulted in the optimal tradeoff between water flux and nitride rejection. Khajouei et al. deduced that for GO loading higher than the optimal concentration, the performance dropped due to the uncertain position of GO functional groups on the membrane surface. Wang et al. used a DFT simulation to understand the mechanism of interaction of pharmaceutical contaminants, triclosan (TCS), and acetaminophen (AAP) with rGO using Vienna ab initio calculations and found that the most stable adsorption configuration of AAP corresponded to the interaction between acylamino groups on AAP and hydroxyl groups on rGO with a calculated binding energy of 0.62 eV. Similarly, the most stable adsorption configuration on TCS corresponded to the interaction between hydroxyl groups on TCS and hydroxyl groups on rGO with a binding energy of 0.56 eV. This shows that AAP has a higher affinity than TCS to the rGO surface, which is consistent with filtration experiments of rGO layers on the PVDF membrane (adsorption capacity of AAP and TCS: 0.023 ${\mathrm{mmol}\;g}^{-1}$ and 0.014 ${\mathrm{mg}\;g}^{-1}$, respectively) [234]. A short summary on the evaluation of foulant and PA membrane interactions using computational studies have been shown in Table 5.

Another efficient and sustainable method is the grafting of functional polymer bushes onto or from the surfaces of polymer substrates or nanostructures. This leads to unique morphologies, thereby exposing functional groups that can readily bind to contaminants. The covalent grafting of the polymers to the nanostructured scaffold results in reduced leaching and higher chemical stability for prolonged usage. For example, Ha et al. grew polystyrene brushes from CNT surfaces by synthesizing vinyl-group-functionalized CNTs and performing in situ polymerization in the presence of an initiator [251]. Similarly, poly(acrylic acid) (PAA) brushes were grafted onto the PVDF membrane using a physisorbed free radical polymerization technique, and Ag NPs were immobilized on PAA imparting hydrophilicity, anti-organic fouling, and anti-biofouling properties to the PVDF membrane [252]. Recently, Ouyang et al. developed an attractive membrane where they grafted an amphiphilic polymer, PVP, onto the surface of the hydrophobic PP membrane. This membrane had a water flux of 50,000 ${L\;m}^{-2}h^{-1}{\mathrm{bar}}^{-1}$ with an adsorption capacity of 18.5 ${\mathrm{mg}\;m}^{-2}$ (42.6 ${\mathrm{mg}\;g}^{-1}$) for the emerging contaminant bisphenol A (BPA). Regeneration and reusability studies showed that the removal capacity remained above 94% even after 10 adsorption/desorption cycles of BPA [253]. Sahu et al. fabricated short polymer brushes of anion exchange resins of vinylbenzyl trimethylammonium chloride and covalently functionalized it onto different carbon nanostructures (SWCNT, fluorographite) in aqueous media that gave rise to conformally coated pinhole-free mesoporous architecture and partially exfoliated stacked nanoplatelet-like thin films that delivered a flux capacity of 692 ${L\;m}^{-2}h^{-1\;}$ and 1100 ${L\;m}^{-2}h^{-1\;}$, respectively. Functionalized SWCNT demonstrated a maximum adsorption capacity of 139 ${\mathrm{mg}\;g}^{-1}$ for sodium fluorescein and functionalized fluorographite removed 99% of the emerging contaminant perfluorooctanoic acid to below 100 parts per trillion, which is close to the health advisory limit set by US EPA [74,75,254].

**Table 5:** A summary of findings of computational studies contributing to understanding foulant interactions, effect of ions, and hydration properties of PA membranes in desalination application.

Membrane Type	Computational Method Used	Modeling Results	Reference
CNT embedded in membrane	Quenched solid DFT to understand effect of foulants, moving particle semi-implicit method to understand implication of foulant (BSA) on velocity and pressure understand foulant (BSA)	Due to fouling, there was decrease in BET surface area (12.63 → 9.77 $m^{2}g^{-1}$), average pore size, and pore volume because of saturated mesoporous structure, foulant content increasing dead flow section, and membrane pressure	[255]
CNT and CNF incorporated in membrane	LAMMPS and OpenMM Ver. 7.5 package to study hydration and permeation with boron as antiscaling contaminant	Simulation demonstrated higher H_2_O diffusion (0.766 × 10^−5^ → 0.923 × 10^−5^ ${\mathrm{cm}}^{2}s^{-1}$) after incorporating CNT and CNF compared to pristine membrane, CNF enhanced water hydration and boron diffusion on the membrane, and CNT responsible for increased charge transfer to PA	[256]
MWCNT incorporated in PA membrane	LAMMPS to study interaction between membrane surface and foulant (BSA)	MWCNT-PA membrane exhibited superior antifouling compared to pristine due to enhanced hydrophilicity, smoother surface, and results in a stiffer PA structure that lowers structural conformity with BSA	[257]
PA and GO membranes	MD simulation to study the effect of presence of ions (Na^+^, Cl^−^) on BSA–membrane interaction	With increase in ionic strength, no changes were observed for protein-PA membrane while repulsion was observed between protein-GO membrane, PA showed attractive interaction with BSA while GO showed a repulsive one	[258]
CNT incorporated in membrane	LAMMPS to study effect of ions and nanomaterials on membrane fouling during crossflow measurements including natural organic matter (NOM) or alginate.	Low MW NOM interacts irreversibly with surface cavities of PA, high MW alginate either uncoil and spread on the surface or bind to foulant via ionic bridge due to Ca^2+^ ions, CNTs induce a stiffer and less rough surface, leading to low conformity to foulant interaction	[259]

BSA: Bovine serum albumin; PA: polyamide; BET: Brunauer–Emmett–Teller; MWCNT: multiwalled carbon nanotubes; CNF: carbon nanofibers; GO: Graphene oxide; MW: molecular weight; LAMMPS: Large-scale Atomic/Molecular Massively Parallel Simulator; DFT: Density functional theory.

### 2.3. Zeolite-Based Nanocomposite

As shown in Table 4, membrane performance is an optimization between selectivity and permeability. Zeolites are a cost effective and green ceramic membrane precursor and have shown the potential to simultaneously have high rejection/selectivity and high-water flux [37,260]. Zeolites are three-dimensional porous crystalline structures of aluminosilicates with primary building units of alumina or silica tetrahedra that organize to form secondary building units that are responsible for the development of unique zeolite properties [261]. Zeolites are classified based on their origin (natural or synthetic), silicon-alumina ratio, pore size, crystal structure and composition, among other factors [262]. Currently, there are more than 70 different types of zeolites and more than 200 modified zeolite frameworks utilized in various engineering applications [260,263,264]. 

The ability of zeolites to contribute to water treatment applications comes from their well-defined porous structure with negatively charged surfaces, voids, and flow channels. These surface charges are balanced by exchangeable ions. Monovalent alkali metal ions and divalent alkaline earth metal ions within the zeolite structure allow for easy ion exchange processes [265,266,267]. Besides metal cations and water molecules in the pores and cavities, other types of molecules and cationic groups can be accommodated as well. For example, there is a selectivity order for the exchange of various cations by clinoptilolite zeolite materials [266]. The silica–alumina ratio in a zeolite is responsible for its chemical stability and the degree of cationic exchange within the zeolite. Zeolites with low or moderate silica content exhibit hydrophilicity, electrostatic interaction with polar molecules, and good adsorption specificity. This gives rise to inter-crystalline defects, which eventually become a tradeoff for selectivity [268]. Zeolites with higher silica content showcase hydrophobicity and are better suited for removing emerging contaminants from drinking water [261]. Depending on the width of the flow channels, zeolites can act as molecular sieves, whereas the width can be tuned by changing the atoms in the framework. Hence, separations in zeolites can occur via competitive adsorption, ion exchange, molecular sieving, or charge exclusion mechanisms [269,270,271].

#### 2.3.1. Naturally Available Zeolites

Clinoptilolites ((K_2_, Na_2_, Ca)_3_Al_6_Si_30_O_72_•21H_2_O, monoclinic) are some of the most abundant and economical zeolites of the heulandite category. They have high crystallinity and have been extensively used for water treatment [272]. Habib et al. impregnated PVC membranes with clinoptilolite NPs (0.5 wt.%) and the morphology analyzed under field emission SEM revealed the presence of larger macrovoids that could facilitate the effective diffusion of water. The incorporation of clinoptilolite in the nanocomposite membrane led to higher hydrophilicity owing to the presence of hydroxyl groups and higher water flux (13.9 → 20.2 ${L\;m}^{-2}h^{-1}$) after 300 min at 0.1 bar pressure, but resulted in a decrease in mechanical strength. The antifouling performance was improved, with a decrease in irreversible fouling rate (19.3 → 6%) and an increase of FRR (80.7 → 94%) for a 0.5 wt.% PVC/clinoptilolite nanocomposite compared to the pristine polymer [273]. Casadellà et al. prepared MMMs by blending different wt.% of clinoptilolite into PSf and PVP matrix for selective recovery of ${\mathrm{NH}}_{4}^{+}$ and $K^{+}$. MMMs with 70 wt.% of clinoptilolite particles showed recovery or desorption capacities of 75% and 60%, respectively, for ${\mathrm{NH}}_{4}^{+}$ and $K^{+}$ with $H^{+}$ ions using ultrapure water at 60 °C [267]. Natural zeolite materials have shown molecular sieving, high selectivity, and high cation exchange capacity, yet water flux is often a performance constraint. Moreover, impurities restrict their exchange efficiency. Many of these limitations can be circumvented using synthetic zeolites [260,274].

#### 2.3.2. Synthetically Available Zeolites

Synthetically produced zeolites have a controlled composition, which can be manufactured at a large scale. Moreover, the structural features of zeolites can be exploited, and the total molecular charge of the zeolite framework can be modified for specific ion diffusion and separation technologies. In the following section, a holistic review of mainly synthetically produced zeolite materials for various water treatment applications are discussed in detail. The incorporation of zeolite into the polymeric matrix allows for enhanced chemical and mechanical stability, permeability, selectivity, adsorption, separation, and desalination processes [275]. 

##### Organic Dyes

**Figure 9:**
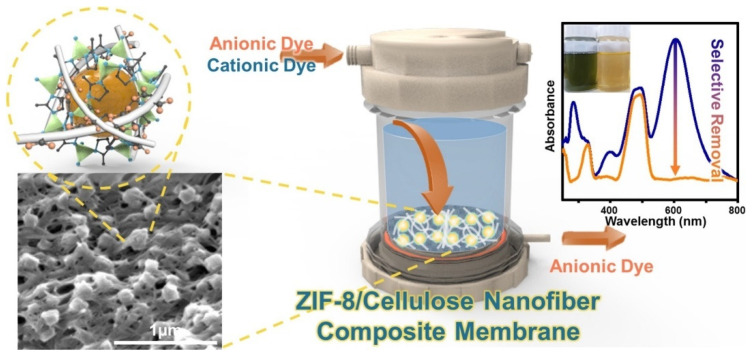
A 21 wt.% ZIF–8 loaded 2,2,6,6-tetramethylpiperidine 1-oxyl radical oxidized cellulose nanofibers membrane. Adapted with permission from Song et al. [279]. Copyright (2019) Elsevier.

Zeolites hold remarkable potential in the removal of toxic organic dyes from wastewater due to their strong electrostatic interactions [276,277,278]. Song et al. prepared a robust cellulose nanofibrous UF membrane by embedding Zeolite Imidazole Framework-8 (ZIF-8, class of metal organic frameworks) as an anchor to hold 2,2,6,6-tetramethylpiperidine 1-oxyl radical (TEMPO) oxidized cellulosic membrane together using an in situ synthesis (shown in Figure 9). It was found out that the 21 wt.% ZIF-8-loaded nanocomposite membrane (20 µm thickness) showed optimum porous structure, with a smaller flux drop (29%) and a higher water flux of 84 ${L\;m}^{-2}h^{-1}{\mathrm{bar}}^{-1}$ as compared to 21 wt.% blend composite and pristine cellulose nanofibers (CNF) with water flux of 11 and 6 ${L\;m}^{-2}h^{-1}$, respectively (after 24 h of filtration (at 1–3 bar)). These membranes showed highly selective removal of cationic dyes (Janus Green B, 98.9%; MB, 93.8%) compared to negatively charged and neutral dyes due to electrostatic interactions with the negatively charged nanofibers [279].

**Figure 10:**
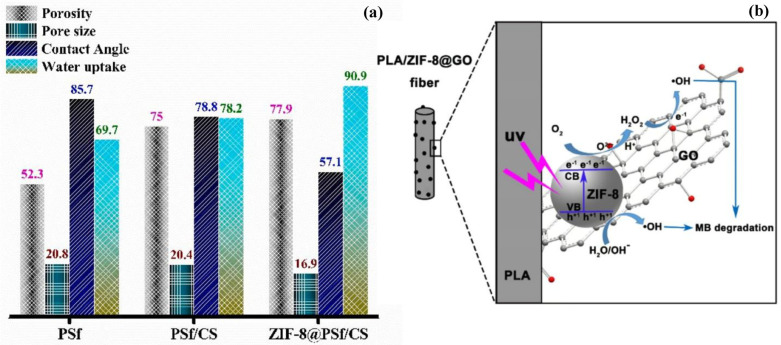
Integration of new membrane properties due to ZIF–8 addition: (**a**) Enhanced hydrophilicity in ZIF–8 modified membrane [280], © Elsevier, 2022; (**b**) Possible mechanism for ZIF–8 mediated photocatalytic degradation of methylene blue [69], © ACS Omega, 2018.

Gowriboy et al. fabricated nanocomposite membranes by blending ZIF-8 NPs with PSf and CS, which resulted in enhanced crystallinity, hydrophilicity (WCA, 85.7° → 57.1°), surface area (580.94 $m^{2}{\;g}^{-1}$), thermal, and mechanical stability. This membrane demonstrated removal of both cationic and anionic dyes (MB, 94.11%; RhB, 94.01%; Acid blue, 86.6% and Congo Red (CR), 85.50%) due to π-π, hydrogen bonding, and electrostatic interactions. The trend of enhancement in porosity and hydrophilicity of these membranes can be observed in Figure 10a [280]. Nanocomposite RO membranes were fabricated by Kim et al. from amino groups carrying sulfonated poly(arylene ether sulfonate) and aminated EMT type zeolite NPs. These membranes exhibited excellent chlorine resistance as evidenced by a negligible reduction in salt rejection (98.8%) and increment in water flux (37.8 ${L\;m}^{-2}h^{-1}$) by 12.7% and 2.5 ${L\;m}^{-2}h^{-1}$, respectively, after the chlorination test [281]. Dai et al. fabricated a membrane by electrospinning poly(lactic acid) and ZIF-8 loaded GO (ZIF-8@GO) and analyzed for hydrophilicity along with MB photocatalytic degradation. The enhanced hydrophilicity was due to the presence of large numbers of hydroxyl and carboxyl groups on the surface of GO. The mechanism of photocatalytic degradation was due to excitation and transfer of electrons from organic ligands of ZIF-8 to GO that react with O_2_ to produce O_2_^•−^. This radical anion can react with H^+^ to produce H_2_O_2_ and subsequent side reactions generate OH^•^ radicals (Schematic shown in Figure 10b). These highly reactive species cause the photocatalytic degradation of MB (90%), even at low concentrations of ZIF-8@GO (0.06 ${\mathrm{mg}\;\mathrm{mL}}^{-1}$) [69].

##### Heavy Metals

**Figure 11:**
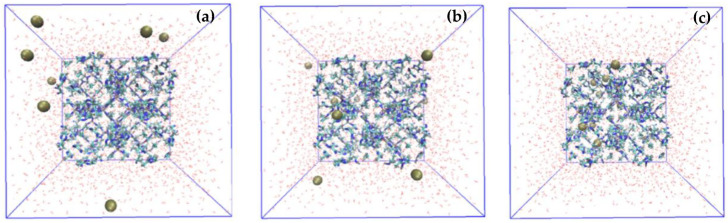
Migration behavior of Cu^2+^ on the ZIF–8 framework (**a**–**c**). Adapted with permission from Li et al. [286]. Copyright (2022) Elsevier.

Heavy metals are a growing concern for environmental pollution due to the rapid growth of industrialization, agriculture, and urbanization [282]. Over the past decade, consistent efforts have been made to modify the surface of zeolites so that they not only possess cation exchange properties but also provide high capacity and selective adsorption [283]. ZIF-67 NPs loaded carboxylated GO sheets were impregnated in PSf hollow fibrous membranes, which were used for Cu^2+^ and Pb^2+^ removal. These membranes demonstrated a Langmuir adsorption isotherm with an excellent water flux of 346 ${L\;m}^{-2}h^{-1}$ and FRR of 95.7%. Adsorption capacities of these membranes for Cu^2+^ and Pb^2+^ were 66.4 ${\mathrm{mg}\;g}^{-1}$ and 86.4 ${\mathrm{mg}\;g}^{-1}$, respectively, and contaminated water testing resulted in 94.5% and 97.8% rejections, respectively, without significant loss from regeneration cycles [284]. Qiu et al. fabricated a TFNC membrane by embedding polydopamine-modified ZIF-8 in a crosslinked matrix generated by poly(ethyleneimine) (PEI) and 1,3,5-benzenetricarboxylic acid chloride. This FO membrane exhibited a 95.8% rejection and a 5.95 ${L\;m}^{-2}h^{-1}{\mathrm{bar}}^{-1}$ water permeability for 5.0 mM of MgCl_2_ under 1.0 bar where this highly selective rejection of MgCl_2_ decreased with increased loading of polydopamine-modified ZIF-8. This is mostly due to the Donnan exclusion effect, resulting in repulsion between divalent cations and the positively charged surface due to PEI. However, the water flux increased with NP loading due to the optimal interface voids generating continuous channels, an increase in pore size, and positive compatibility between ZIF-8 and PA matrix. This membrane exhibited remarkable FO mode rejection of heavy metal ions (Cu^2+^, 99.1%; Ni^2+^, 98.3%; Pb^2+^, 97.7%) [285]. Li et al. fabricated a UF membrane by electrospinning ZIF-8 with PAN solution and demonstrated adsorption efficiencies of 89%, 92%, and 76% for CR, Pb^2+^, and Cu^2+^, respectively. Adsorption mechanisms of these contaminants were investigated by DFT calculations and MD simulations. It was found that all the contaminants easily adsorb on the ZIF-8 surface via physisorption. In addition to this, some frameworks collapsed due to release of Zn^2+^ due to Pb^2+^ adsorption, meaning ZIF-8 takes up an ion exchange role, resulting in chemisorption for Pb^2+^. MD simulations investigated the dynamic approach of Cu^2+^ at ZIF-8 surface and revealed that under 5 ns, all Cu^2+^ ions migrated into ZIF-8 due to interactions between Cu^2+^ and carbon and oxygen atoms; within 20 ns, dynamic equilibrium is achieved (shown in Figure 11) [286]. A comprehensive overview of zeolite–polymer nanocomposite membranes used for the removal of heavy metals and other molecules/ions are shown in Table 6.

**Table 6:** An overview of nanocomposite membranes for the effective removal of especially heavy metal ions and few inorganic ions and molecules.

Nanocomposite Membrane Composition	Heavy Metal Ion (or Other Molecules/Ions)	Adsorption Capacity (mg g^−1^) (or Recovery/ Removal Rate ^b^ (%))	References
Incorporation of NaX zeolite NPs into PSf membrane	Ni(II), Pb(II)	122.0, 682.0	[265,287]
Impregnation of zeolite and PVP in matrix of PSf	Cu(II)	38	[288]
Hybrid membrane made up of Ca-Activated zeolite, PVP, and PES blend	${\mathrm{PO}}_{4}^{3-}$	70 ^b^	[289]
Fabricating ZIF-8 NPs into cellulose UF membranes	As(III), methylene blue	97.7, 100 ^b^	[71]
Mixing of zeolite into chitosan (CS) and poly(vinyl alcohol) PVA mixture via electrospinning	Cr(VI), Fe(III), Ni(II)	8.84, 6.16, 1.77	[290]
Mixture of Polycaprolactone and clay was electrospun	Cd(II), Cr(III), Cu(II), Pb(II)	29.59, 27.23, 25.36, 32.88	[291]
Mixture of PVA and clay was electrospun		14.58, 17.36, 16.46, 16.50	
Integrating ZIF-8 NPs into PAN UF membrane	Congo Red, Pb(II), Cu(II)	89, 92, 76 ^b^	[286]
Embedding zeolite and PVP into PSf matrix	Cu(II)	96.4 ^b^	[292]
Blending of zeolite into CS and PVA mixture via casting	Cr(VI)	450	[293]
Incorporating NaX zeolite into PVA via electrospinning	Ni(II), Cd(II)	342.8, 838.7	[294]
Pd growth on electrospun mat of zeolite and poly-acrylonitrile-co-methyl acrylate using electroless plating	Ammonia nitrogen (NH_4_^+^-N)	92 ^b^	[295]
Deposition of microfine powdered zeolite on outer surface of PVDF fiber membrane	Total organic carbon, total nitrogen, NH_4_^+^-N	~18, ~20, ~90 ^b^	[296]

b: Recovery or removal rate in the third column can be identified by values with b superscript.

##### Desalination

Although TFCs usually consist of a PA layer on top with a porous PSf substrate to ensure higher permeability than the commercially available cellulose triacetate membranes [297], the loading of zeolite NPs in TFNC membranes has further enhanced the water permeability and desalination rate [298]. Zeolite materials have been extensively used to fabricate TFNC from TFC membranes by impregnating NPs in the active rejection layer of PA or into the PSf substrate to optimize RO and FO specific applications. Zeolite-loading-based membrane performance is an interplay between the interfacial polymerization process, surface roughness, and voids or flow channels due to substrate and active layer interactions that endow these membranes with new properties. These modifications yield enhanced water flux, solute flux, and salt rejection. Cay-Durgun et al. performed long term (3000 h) performance testing of NPs-loaded PSf TFNC membranes that exhibited enhanced water permeability, salt rejection, and hydrophilicity (details in Table 7) [299]. Ma et al. performed RO and FO tests on a NaY zeolite-loaded TFNC membrane, and for FO tests, the active-layer-facing-draw solution (AL–DS) and active-layer-facing-feed solution (AL–FS) orientations were evaluated. In order to minimize internal concentration polarization, which significantly reduces water permeability, a structural parameter denoted as S (thickness × tortuosity/porosity) and the hydrophilicity of membrane substrate were taken into consideration in FO tests. A lower value of S is required for superior water flux performance, which indicates lower tortuosity, thinner structure, and higher porosity [300,301]. B/A is the ratio of solute permeability to water permeability, which is important in the selectivity process of FO membranes, where a small B/A value means reduced solute back diffusion [301,302,303,304]. Table 6 summarizes the enhancement in characteristics and performance of FO/RO specific TFNCs compared to TFCs (or pristine membranes) due to the inclusion of zeolite particles.

Overall, the high thermal and chemical stability as well as the tunable porous structure make the zeolite system ideal for high water flux treatment applications. Moreover, the ion exchange and molecular sieving properties make zeolites and related frameworks promising alternatives for molecule/ion removal, recovery, and desalination [274].

### 2.4. Biopolymer-Based Nanocomposites

Biopolymer nanocomposite technologies have received significant interest in recent years due to their promising applications as sustainable water purification membranes. However, research on these materials is mostly limited to MF, UF, or NF applications [307]. Although biobased polymeric membranes have been widely researched, issues related to structural robustness, high-capacity removal, resistance to external conditions, and long-term performance are salient features that need to be carefully assessed. Xie et al. reconstituted aquaporin Z (channel proteins) into self-assembled poly(2-methyl-2-oxazoline)-block-poly-(dimethysiloxane)-block-poly(2-methyl-2-oxazoline) vesicles and immobilized the protein onto a porous cellulose acetate membrane to enhance the membrane stability. For the membranes incorporated with aquaporin Z, the salt rejection rose from ~0% in the control sample to 61% and 75% for NaCl and MgCl_2_, respectively. These membranes demonstrated a water flux of ~23 ${L\;m}^{-2}h^{-1}{\mathrm{bar}}^{-1}$ while maintaining a high mechanical strength, proving to be a promising candidate for NF or FO application [308]. This section will shed light on some of the developments on biodegradable cellulose-based and chitosan (CS)-based membranes leading towards advanced water and wastewater treatment applications.

#### 2.4.1. Cellulose-Based Nanocomposites

Cellulose, the world’s most abundant biopolymer, has been extensively studied for both its multitude of facile modification mechanisms and its incorporation into water and wastewater treatment systems. The first RO membranes were cellulose acetate based [309]. The primary C-6 and the secondary C-2 and C-3 hydroxyl groups on the D-glucose monosaccharide units allow a variety of reactive mechanisms to be employed [310,311,312,313]. To be used for water purification and treatment, cellulose has been modified using esterification [314], etherification [315], halogenation [316], phosphorylation [317], xanthation [318], oxidation [319], sulfonation [320], carboxymethylation [321], hydrolysis [322], nitro-oxidation [323], and polymer surface functionalization [324,325] methods. These modifications can help fine tune their affinity towards specific contaminates and/or add beneficial features [314,324,326,327,328,329].

**Figure 12:**
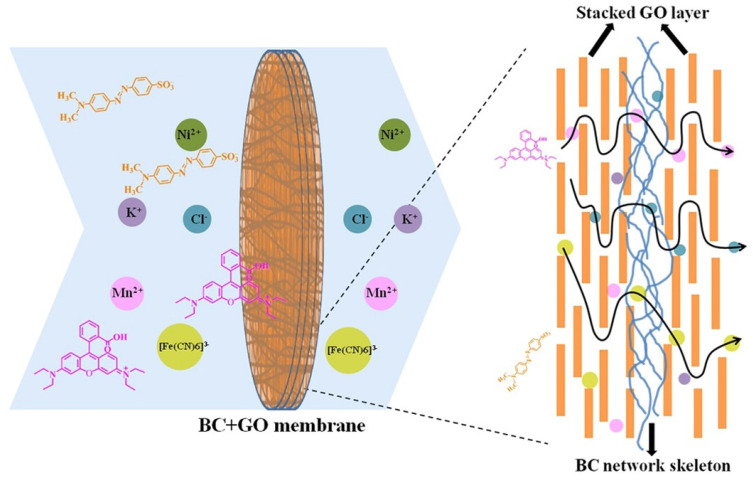
Mechanisms of free–standing bacterial cellulose and graphene oxide membrane for selective ion permeation. Adapted with permission from Fang et al. [70]. Copyright (2016) Scientific Reports.

Interest in cellulosic nanomaterials is due in part to their high abundancy, low cost, biodegradability, sustainability, thermal stability, high flexibility, low density, and mechanical strength [325,330,331,332,333]. However, despite their environmental benefits due to their biodegradability, the long-term performance of these membranes remains a problem as they are susceptible to bacterial breeding, which needs to be improved. In a study by Xu et al., cellulose nanocrystals (CNC)/Ag were embedded into the PA layer of TFNC NF membrane, and 0.01 wt% CNC/Ag loading resulted in high water permeability (25.4 ${L\;m}^{-2}h^{-1}{\mathrm{bar}}^{-1}$), a high NaSO_4_ rejection rate (99.1%), and remarkable antifouling (FFR: 92.6% for humic acid) and antibacterial activity (reduction in *E. coli* viability: 99.4%) [334]. Though CNCs are usually processed by the acid hydrolysis method [335], a plant-based or green-chemical-based modification is of prospective research interest. Zhang et al. fabricated a nanocomposite membrane incorporating Ag-loaded TiO_2_ NPs, CNF, and CS that was capable of bacterial eradication (*E. coli*, 99.97%; *B. subtilis*, 99.98%; *S. aureus*, 99.98%), high oil–water emulsion (~98.5%), and high MB photodegradation (96.25%) [336]. Yang et al. formulated clean water remediation by forming copolymers of tobramycin and dopamine through mussel mimicked polymerization (PDA/TOB) and coated these PDA/TOB NPs on a cellulose acetate membrane. These highly durable membranes exhibited remarkable photothermal efficiency and antibacterial properties, which were used for solar mediated steam generation. These low-cost membranes exhibited an evaporation rate of 1.61 ${\mathrm{kg}\;m}^{-2}h^{-1}$ and evaporation efficiency of 92.4% under 1 sun irradiation [337]. Chemically or mechanically modified cellulosic nanomaterials have been reported to have a remarkably high affinity towards various heavy metals from drinking water. These include Cd(II), Cu(II), Pb(II), Hg(II), Ni(II), Cr(III) [315], Ag(I), Co(II), As(V), and Zn(II) [338]. For cellulosic materials, the two main mechanisms involved in the removal of heavy metal ions are ion exchange and chemical complexation mechanisms [339]. Cellulose materials possess excellent hydrophilicity due to the presence of -OH groups on their surface, making them perfect additives for TFNC membranes [340]. Hoang et al. deposited cellulose nanocrystals (CNC) as an interlayer on PES substrate and a barrier layer was fabricated using the IP method. For a loading of 5.5 ${\mathrm{mg}\;\mathrm{cm}}^{-2}$ of CNCs, the water flux (23.92 ${L\;m}^{-2}h^{-1}$) was increased by >70% compared to the pristine TFC membrane and demonstrated exceptional removal efficiency for toxic heavy metals (CuSO_4_, 98%; CuCl_2_, 96.5%; PbCl_2_, 90.8%) [341]. A selective ion permeation membrane was prepared by vacuum filtration of a mixed solution of bacterial cellulose (BC) and GO, where porous BC layers were intercalated between GO sheets that added to the stability and tensile strength of these membranes. These membranes operated on the size exclusion principle where large molecules like RhB and MO are rejected and small (hydrated radii < 1 nm) inorganic ions (Ni^2+^, Mn^2+^, Cl^−^, [Fe(CN)_6_]^3−^) permeate through it at a rate inversely proportional to their size (Figure 12) [70].

TEMPO-oxidized cellulose nanofibers (CN) were embedded within stacked GO sheets and the dispersion was coated onto a PVDF membrane to obtain MMMs with high water permeability (816 ${L\;m}^{-2}h^{-1}{\mathrm{bar}}^{-1}$) and efficient removal (82–99%) of neutral, anionic, and cationic dyes. They performed reactive MD simulation calculations to understand the mechanism of rejection, which revealed that the dyes exhibited adsorption behavior based on H-bonding, π-π stacking interactions between dye molecules and GO planes, and self-assembly [342]. Ma et al. incorporated CN and MWCNT in the PVA barrier layer of TFNC composed of a PAN scaffold mid-layer and polyethylene terephthalate non-woven substrate for UF applications. Compared to commercial PAN_10_ UF membranes, they achieved 10 folds higher permeation flux by adding nanofillers without compromising the rejection ratio (~99.5%) [343].

The dispersion of cellulosic nanomaterials in hydrophobic polymer membranes is one of the ongoing challenges associated with these materials. However, it has been reported that surface grafting with polymers can potentially be a solution [339]. Nazri et al. incorporated microcrystalline cellulose (MCC) into hydrophobic PES matrix using the NIPS method and found that 3 wt.% MCC resulted in improved water permeability (51.50 ${L\;m}^{-2}h^{-1}{\mathrm{bar}}^{-1}$) compared to the pristine membrane and a 96.14% humic acid rejection. Although the inclusion of MCC results in a better PI rate, leading to elongated and bigger pore sizes benefiting the flux response, it is important to note that 3 wt.% loading also reduced the tensile strength (6.57 → 5.71 MPa) compared to the pristine membrane due to an aggregation issue in the casting solution [340]. Other studies have shown better flux responses, 485 ${L\;m}^{-2}h^{-1}{\mathrm{bar}}^{-1}$ [344] and 692 ${L\;m}^{-2}h^{-1}{\mathrm{bar}}^{-1}$ [345], when using CNC and lignin cellulose nanofibrils in the PES membrane in the presence of PVP additive, which adds to the pore-forming property and reduce the aggregation of NPs.

#### 2.4.2. Chitosan-Based Nanocomposites

Much like cellulosic nanomaterials, advantages such as low cost, high abundancy, reactivity, high hydrophilicity, biodegradability, and biocompatibility make CS nanocomposites a subject of interest in the current fields of water and wastewater treatment [346,347]. CS has a similar structure to cellulose, with C-2 acetamido groups and amine groups replacing the C-2 secondary hydroxyl groups. This allows for an abundance of amino and hydroxy groups, which can chelate with positively charged metal ions, cationic molecules, and negatively charged metal oxyacid ions via electrostatic interactions; therefore, it is widely used in heavy metal removal [217,348,349,350]. CS has been employed in various forms for water treatment, such as NPs [351], fibers [352], coatings [353], flakes [354], nanorods [355], membranes [356], and hydrogels [357], among others. However, pristine CS possesses low thermo-mechanical properties, low porosity, and poor stability, and therefore requires reinforcement to enhance the membrane’s mechanical strength [358,359]. Hydroxyapatite was introduced in the CS solution to form electrospun nanocomposite membranes because pristine CS renders low flexibility, high viscosity, and poor mechanical properties, making it difficult to handle. These membranes delivered an exceptional adsorption capacity of 296.7, 213.8, and 180.2 ${\mathrm{mg}\;g}^{-1}$ for heavy metal ions Pb^2+^, Co^2+^, and Ni^2+^, respectively, within 30 min equilibrium time at 45 °C. The sorption followed pseudo second order kinetics and a Langmuir adsorption isotherm [348]. Aliabadi et al. electrospun polyethylene oxide and CS solution, which formed sorption-selective nanocomposite membranes for heavy metals in the order: Ni^2+^ (175.1 ${\mathrm{mg}\;g}^{-1}$) > Cu^2+^ (163.7 ${\mathrm{mg}\;g}^{-1}$) > Cd^2+^ (143.8 ${\mathrm{mg}\;g}^{-1}$) > Pb^2+^ (135.4 ${\mathrm{mg}\;g}^{-1}$). These nanofibers exhibited an average surface area of 312.2 $m^{2}g^{-1}$ using BET analysis. Regeneration and reusability studies for 5 cycles showed gradual reduction in capacity due to the loss of active sites during acid regeneration [347]. In another study, CNCs with functional groups (SO^3−^ and/or COO^−^) were incorporated in the CS matrix via the freeze-drying method and cross-linking was performed using glutaraldehyde vapors. This membrane was successfully used for the removal of the positively charged dyes (Victoria Blue 2B, 98%; methyl violet 2B, 84%; Rhodamine 6G, 70%) after 24 h and demonstrated a water flux of 64 ${L\;m}^{-2}h^{-1}$. CNC was not only used as a reinforced nanofiller, but also provided functional sites for high capacity adsorption [360].

CS has been employed to form nanocomposite membranes using solvent casting, solvent evaporation, and electrospinning techniques to obtain the desired porosities and targeted functionalities for specific adsorption. Gharbani et al. fabricated PVDF/g-C_3_N_4_/CS membrane via dissolution casting and demonstrated a removal rate of 72.74% for an initial concentration of 2 ${\mathrm{mg}\;L}^{-1}$ RhB under a pH of 3 and 3% CS loading [361]. Huo et al. fabricated a sustainable acid-resistant CNF/CS membrane using the solvent casting and solvent evaporation method and demonstrated anionic MO removal with an adsorption capacity of 655.23 ${\mathrm{mg}\;g}^{-1}$ due to H-bonding, charge interaction, and n-π stacking interaction with no affinity towards cationic dyes (MB and MG). This membrane demonstrated a slight reduction (98.50 → 89.65%) in MO adsorption in reusability experiments after six cycles [362]. Wu et al. fabricated an electrospun porous support layer of CS/PVP/PVA, on which an active layer coating of SWCNT/CS/PVP was performed using the electrospray method, and was finally crosslinked by glutaraldehyde vapors. Optimized UF thin-film membranes exhibited a water flux of 1533.26 ${L\;m}^{-2}h^{-1}$ comparable to a commercial PVDF UF membrane, a high dye rejection (MG, 87.20%; MB, 76.33%; CV, 63.39%), heavy metal removal (Cu^2+^, 95.68%; Ni^2+^, 93.86%; Cd^2+^, 88.52 %; Pb^2+^, 80.41%), and enhanced antifouling properties [217]. Montaser et al. developed antimicrobial activity in CS by reacting CS with salicylaldehyde as a crosslinker using Schiff base reaction which resulted in salicylimine-functionalized CS that formed a metal complex with TiO_2_ NPs to form the nanocomposite membrane. These membranes demonstrated full bacterial eradication of *S. aureus* and *P. aeruginosa* at two different concentrations (0.25 × 10^−2^ and 0.5 × 10^−2^ ${g\;\mathrm{mL}}^{-1}$). The tensile strength and elongation were enhanced due to the integration of TiO_2_ NPs [363]. Yu et al. fabricated a membrane consisting of modified cellulose acetate, modified CS, and TiO_2_ for oil–water separation and Cu^2+^ adsorption. In order to improve the heavy metal adsorption capacity of these membranes, the amino groups in CS were modified into N-salicylic groups using the Schiff base. This membrane exhibited 99.4% oil–water separation efficiency for cyclohexane. At neutral pH, the adsorption capacity for Cu^2+^ was 220.67 ${\mathrm{mg}\;g}^{-1}$ and for a concentration of 1000 mg·g^−1^, the adsorption efficiency was 97% [364]. Habiba et al. electrospun a nanofibrous composite of CS/PVA/zeolite that exhibited a 100% increase in Young’s Modulus because of the incorporation of zeolite and an adsorption capacity of 153 ${\mathrm{mg}\;g}^{-1}$ for MO dye [365].

By 2025, more than 20,000,000 end-of-life RO membranes will be generated globally per year [307]. Therefore, using biopolymers for RO techniques will alleviate the waste and its environmental impact. However, fouling of these biobased membranes is one of the challenging issues while addressing membrane performance. Hegab et al. generated a layer of chemically functionalized CS with GO by forming amide bonds between carboxylic groups and amino groups of GO and CS, respectively. This layer was fabricated on a TFC PA membrane, which was tested against fouling resistance using BSA. This functionalized membrane exhibited enhanced permeation flux (56.1 → 61.5 ${L\;m}^{-2}h^{-1}$), salt rejection (88.7 → 95.6%), and FRR (86 → 97%) compared to the pristine PA layer [366].

## 3. Summary, Impact, and Future Scope

**Figure 13:**
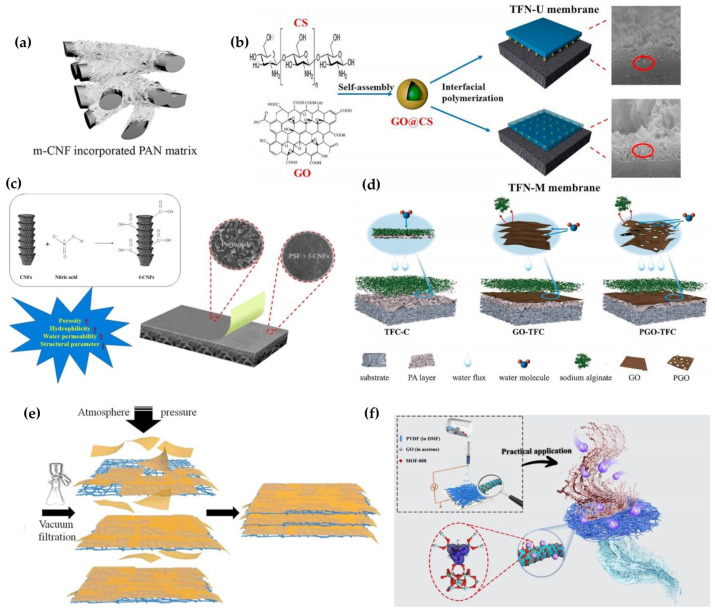
Various approaches of fabricating polymers and nanomaterials into membranes for water treatment applications: (**a**) cysteine-grafted cellulose nanofibers impregnated in electrospun polyacrylonitrile scaffold (microfiltration) [384], © Elsevier, 2014; (**b**) Graphene oxide (GO)-coated chitosan nanoparticles incorporated into (TFN-M) or at the bottom (TFN-U) of polyamide (PA) layer during interfacial polymerization process (ultrafiltration) [64], © Elsevier, 2021; (**c**) carboxylated carbon nanofibers embedded into polysulfone layer via phase inversion process with PA layer on top (forward osmosis) [385], © Elsevier, 2020; (**d**) GO coated on PA layer via layer-by-layer technique [386], © Elsevier, 2022; (**e**) Vacuum filtration of bacterial cellulose and GO dispersion [70], © Scientific Reports, 2016; (**f**) Membrane based on electrospun fibers of homogenous slurry of polyvinylidene difluoride and GO mixed with metal organic framework [67], © Elsevier 2022.

This review summarizes the state-of-the-art as well as comprehensive advances in the integration and distributions of various NPs, with morphologies that optimize the performance of nanocomposite membranes used for water treatment applications. Various fabrication techniques in terms of loading positions of NPs and types of polymeric- or NP-based TFNC membranes are summarized in Figure 13. These membranes target emerging contaminants in the form of toxic molecules or ions in purified drinking water and wastewater. For example, several polymer nanocomposite studies have been conducted using different NPs such as silica NPs [367], nanoscale zero-valent iron [368], poly(piperazineamide) [369], selective polyamide layer [370], montmorillonite [371], nano-sized MoS_2_ [372], GO [373], MWCNT [374], cellulose [324], etc., to achieve the efficient removal of PFAS, one of the proposed emerging contaminants by the US EPA. In addition to removing pollutants, the merits of integrating NPs in membrane filtration technologies includes the addition of structural and chemical properties such as chemical and thermal stability, antifouling, addition of surface charge, mechanical strength, enhanced hydrophilicity, porosity, tunable pore size and permeability, among others [215,375,376]. These nanoadsorbents save time and energy during the water and wastewater treatment processes [375]. Despite their extensive use and high removal capacity, nanomaterials have underlying issues keeping them from widespread applicability. Past studies have shown issues of interface incompatibility between the organic polymeric layer and inorganic NPs [377,378,379,380]. This incompatibility leads to the detachment or leaching of nanomaterials from the membrane surface, which not only affects the efficiency of purification but also leads to secondary environmental contamination [40,65]. Exposure of nanomaterials into the environment, including natural water resources, can result in undesired toxicity and risks that need to be systematically assessed. To minimize leaching, further research is needed on reliable techniques such as covalent attachment, grafting, or cross-linking to enhance the binding between NPs and the polymer matrix. A second issue, due to incompatibility and high surface reactivity, is agglomeration of NPs. Unoptimized membrane fabrication or long-term use can challenge membrane performance through the formation of undesirable voids and cracks [54,381,382,383].

The isolation and reusage of these materials for water treatment or other applications could be a possible solution, which supports a circular economy [387,388,389]. The synthesis and fabrication of adsorption and purification membranes should use green chemicals and/or solvents to allow a relatively benign approach and reduce the possibility of secondary contamination [74,254,390,391]. To alleviate the general environmental contamination problem, the use of nanomaterials with photocatalytic activity can be implemented, which would allow for the breakdown of the extracted contaminants, making the effluent or secondary waste stream free of contamination [375]. However, high operating costs and reliance on UV radiation makes this system inefficient [392]. Additionally, it is necessary to evaluate the various byproducts of the photocatalyzed degradation, and whether this process generates secondary, and possibly worse, contaminants. All of the fabrication techniques employing modifications in PSf membranes are relatively expensive. Therefore, there is a tradeoff between cost efficiency and performance [393]. In order to ensure cost effectiveness and long-term use, regeneration and reusability are important factors to be considered for these nanocomposite membranes [43,54,74,75,393].

There has been significant research performed to design and synthesize novel high-capacity, green, sustainable polymer-based nanocomposite membranes for water and wastewater treatment. Despite these consistent efforts, several obstacles exist due to lack of research studies that can be used as a guide for commercial production [40]. Biodegradable polymeric membranes have been attractive for their ability to harness the hierarchical structural and mechanical properties of naturally produced biomaterials, leaving a vanishingly small human footprint on the environment. Nanomaterials are also designed to reduce the impact on the environment by providing highly efficient and reusable solutions [394]. For example, nanomaterials have been used in automotive exhaust systems to promote reactions that reduce pollution and promote cost efficiency [395]. It is important to harness the positive effects of nanomaterials that can lead to efficient and sustainable TFNC membrane systems. Computational chemistry methods are useful and convenient tools in this case to understand small scale complexities of novel membrane structures, characteristics, and/or performance. This review provides an overview of the extensive research that has been done in laboratories or at the pilot scale on various combinations that can help researchers in selecting the required materials and techniques. The future research scope includes the evaluation of long-term viability with a focus on regeneration and reusability of nanocomposite membranes with real feed solution testing, environmental contamination due to membrane processing, cost efficiency, and scaling up raw material production [40]. To meet the global demand for clean and safe drinking water, these knowledge gaps require further investigative research efforts to improve the understanding of the commercial-scale production of affordable, efficient, and sustainable water and wastewater treatment membranes. 

## Figures and Tables

**Table 1:** Different types of contaminants and their associated adverse health effects, examples, and maximum concentration levels.

Contaminants	Generation Source	Impact on Human Health or Ecology	Examples and Their Maximum Concentration Levels (Parts per Billion (ppb))
Organic	Pesticides, pharmaceuticals, natural organic matter, disinfection byproducts, endocrine disrupting chemicals, hormones and steroids, personal care products, flame retardants, plasticizers [6,10]	Mutagenicity, carcinogenicity [17], bladder cancer, developmental issues, increased birth defects [18]	Dibromochloropropane—0.2, simazine—4 [9], Dioxin (2,3,7,8-TCDD)—0.00003, Hexachlorocyclopentadiene—50, Hexachlorobenzene—1 [19]
Inorganic (acids, salts, heavy metals)	Byproduct of metal mining, smelting, fossil fuel combustion, mineral deposits, anerobic groundwater, soil erosion	Toxic effect on aquatic flora and fauna, catharsis, congenital malformation, increased cancer risk, cardiovascular effects [20]	Arsenic—10, cadmium—5, lead —15 [11], Mercury—2, cyanide —200 [13]
Microbial (bacteria, virus, algae, protozoa)	Human and animal fecal wastes, Fertilizer, livestock, sewage	Typhoid, cholera, diarrhea, damage to liver, skin, nervous system, stomach cramps [21]	*E. coli*—0 [22]
Perfluoroalkylated compounds	Firefighting foams, lubricants, coating additives, cookware, food packaging, textile industry, paper packaging [8]	Adversely affect growth, birth weight, fertility disorders, early menopause, thyroid malfunction, and carcinogenesis [7]	Perfluorooctane sulfonate, perflourooctanoic acid—0.07 (both individually and combined) [14]
Radioactive substances	Mining and processing of radioactive minerals	DNA damage, osteosarcoma incidence, leukemia, stomach cancer, urinary cancer, biomarkers of renal (tubular) damage [23]	Uranium (U)—30 [11,13]

**Table 2:** A summary of enhancements in properties of SiO_2_ nanoparticle-based nanocomposite membranes.

Membrane Type	Enhancements Due to Modification	Reference
Tubular hollow nanofiber PVC membrane with dispersed hydrophobic nano-SiO_2_ for water in oil emulsion separation	High permeation flux, thermal, and hydrophobic stability, outstanding lipophilicity and superhydrophobicity	[168]
MSNs (~500 nm) incorporated in presence of PVP in PSf UF membrane	Enhanced hydrophilicity, methylene blue (MB) rejection (84.7%), but decreased water permeability with increase of MSNs wt.%	[164]
PES-MSNs nanocomposite UF membranes	Higher thermal stability, hydrophilicity, porosity, antifouling, and water uptake properties. Properties deteriorate at highest (4 wt.%) loading due to agglomeration	[134]
Silica NPs grafted onto PHEMA on PES membrane (PES)/SiO_2_-g-PHEMA carboxyl-modified fluorocarbon surfactant functionalized PEG segment: fPEG-COOH; Grafting fPEG-COOH onto surface of the PES/SiO_2_-g-PHEMA forming amphiphilic porous membrane	Higher oil–water flux, flux recovery ratio, lower flux decline ratio, antifouling, and self-cleaning properties	[169]
Composite membrane of Ce-doped nanosilica dispersed in PSf prepared by sol–gel process for oil–water separation	Higher tensile strength, hydrophilicity, and antifouling property, >98% oil retention rate	[170]
Porous MCM-41 silica NPs and nonporous silica incorporated into PA thin-film layer via IP process with PSf support at the bottom	Higher surface hydrophilicity, water flux/permeability compared to nonporous structure, enhanced salt rejections (NaCl (98.1%) and Na_2_SO_4_ (98.6%))	[171]
Incorporation of fumed silica NPs functionalized with APTES into chloromethylated PSf matrix using vapor induced phase inversion and NIPS processes	High water permeance (0.46 ${L\;m}^{-2}h^{-1}{\mathrm{bar}}^{-1}$) and high percentage removal of contaminants (reactive red (99.99%), direct yellow (99.94%), methyl green (99.80%), rhodamine B (99.79%), crystal violet (98.69%). Negative impact on mechanical and selectivity for 3 and 4 ${\mathrm{mg}\;g}^{-1}$ loading due to agglomeration.	[80]

PVC: polyvinyl chloride; MSNs: mesoporous silica; PES: polyether sulfone; PEG: poly(ethylene glycol); PSf: polysulfone; UF: ultrafiltration; PA: polyamide; PHEMA: poly (2-hydroxyethyl methacrylate); APTES: (3-aminopropyl)triethoxysilane; IP: interfacial polymerization; PI: phase inversion; NIPS: non-solvent-induced phase separation; NPs: nanoparticles.

**Table 7:** Comparison of zeolite-loaded TFNC membranes with pristine membranes under specific conditions.

Nanocomposite Membrane	Operating/Working Conditions	Results		References
Incorporation of NaY zeolite NPs into the PA layer on porous PSf TFNC membrane	RO tests: 500 ${\mathrm{mg}\;L}^{-1}$ NaCl feed solution under 2.5 bar	0.1% (wt./v) loaded	0.4% (wt./v) loaded	[305]
		Enhanced water permeability: (4.0 × 10^−12^ → 7.1 × 10^−12^ ${m\;\mathrm{Pa}}^{-1}s^{-1}$), reduction of salt rejection (95.6 → 77.6%), exacerbation in B/A (9.74 → 61.1 kPa)	Decreased water permeability (4.13 × 10^−12^ ${m\;\mathrm{Pa}}^{-1}s^{-1}$), improved salt rejection (90.5%), improvement in B/A (22.2 kPa)	
	FO tests: Both FS and DS at 500 mL min^−1^ cross flow rate FS: 10 mM NaCl or DI DS: 0.5, 1.0 or 2.0 M NaCl	S value (782 ± 160 µm) comparable to Hydration Technology Inc. FO membranes For DS: 1.0 M NaCl, FS: 10 mM NaCl and 0.1% (wt./v) loaded TFNC combination: ~50% enhanced water flux in AL-DS (30.7 ${L\;m}^{-2}h^{-1}$), ~50% enhanced water flux in AL-FS (14.6 ${L\;m}^{-2}h^{-1}$) For DS: 1.0 M NaCl), FS: DI water and 0.2% (wt./v) loaded TFNC combination: ~100% enhanced solute flux in AL-FS, >100% enhanced solute flux in AL-DS		
Incorporation of 0.30 wt.% LTA zeolite NPs in PA layer on PSf TFNC membrane	Long term test (3000 h) under 200 psi	Enhanced water permeance (3.7 → 5.3 ${µm\;\mathrm{MPa}}^{-1}s^{-1}$), enhanced salt rejection: (97.4 → 97.9%), improved contact angle before test (62.1 → 95.2°), improved contact angle after test (44.0 → 50.8°)		[299]
Incorporation of NaY zeolite NPs in the PA layer on porous PSf TFNC membrane	Optimal compatibility at 0.5 wt.% loading	Lower S value (0.34 mm) compared to conventional TFNC FO membranes (0.96 mm), enhanced water permeability (128 → 461 ${L\;m}^{-2}h^{-1}{\mathrm{bar}}^{-1}$), enhanced hydrophilicity (contact angle, 53 → 50°)		[298]
	FO tests: Both FS and DS at 500 ${\mathrm{mL}\;\min}^{-1}$ cross flow rate FS: 10 mM NaCl or DI DS: 0.5, 1.0 or 2.0 M NaCl	For DS: 0.5 M NaCl, FS: DI and 0.5 % (wt./v) loaded TFNC, >100% enhanced water flux in AL-DS (43 ${L\;m}^{-2}h^{-1}$), >100% enhanced water flux in AL-FS (21 ${L\;m}^{-2}h^{-1}$) For DS (2.0 M NaCl), FS (DI water), and 0.5 % (wt./v) loaded TFNC, highest FO water flux reported under similar conditions (86 ${L\;m}^{-2}h^{-1}$)		
Incorporation of surface-modified clinoptilolite into PSf substrate by phase inversion method and coating of PA layer on top	Optimal compatibility at 0.4 wt.% loading	Enhanced surface porosity (80 → 85.4%), better water permeability (118.2 → 185.3 ${L\;m}^{-2}h^{-1}{\mathrm{bar}}^{-1}$), lower S value (0.78 → 0.48 mm), enhanced hydrophilicity (contact angle, 71.45 → 57.24°) (surface of clinoptilolite modified with hexadecyl trimethyl ammonium bromide to enhance hydrophilicity)		[306]
	RO tests: 20 mM NaCl aqueous solution at 2.5 bar	For 0.4 wt.% loading, enhanced water permeability (1.93 → 2.74 ${L\;m}^{-2}h^{-1}{\mathrm{bar}}^{-1}$), exacerbation in B/A value (9.86 → 13.99 kPa), slightly reduced salt rejection (96.2% → 94.7%)		
	FO tests: FS: 10 mM NaCl DS: 0.5 or 2.0 M NaCl	FO performance for 10 mM NaCl as FS and 2 M NaCl as DS in AL-DS orientation (for 0.4 wt.% loading): ~50 % enhanced water flux in AL-DS (33.1 ${L\;m}^{-2}h^{-1}$), >50% enhanced water flux in AL-FS (~24.1 ${L\;m}^{-2}h^{-1}$), >100% enhanced solute flux in AL-FS (~15 ${L\;m}^{-2}h^{-1}$), ~100% enhanced solute flux in AL-DS (~20 ${L\;m}^{-2}h^{-1}$)		

RO: reverse osmosis; FO: forward osmosis; AL-DS: active-layer-facing-draw solution; AL-FS: active-layer-facing-feed solution.

## Data Availability

Not applicable.
